# Analysis of the structure and interactions of the SARS-CoV-2 ORF7b accessory protein

**DOI:** 10.1073/pnas.2407731121

**Published:** 2024-11-07

**Authors:** Minh-Ha Nguyen, Gyula Palfy, Marie-Laure Fogeron, Martí Ninot Pedrosa, Johannes Zehnder, Vaclav Rimal, Morgane Callon, Lauriane Lecoq, Alexander Barnes, Beat H. Meier, Anja Böckmann

**Affiliations:** ^a^Molecular Microbiology and Structural Biochemistry, Unité Mixte de Recherche 5086 CNRS/Université de Lyon, 69367 Lyon, France; ^b^Department of Chemistry and Applied Biosciences, Institute of Molecular Physical Sciences, Eidgenössische Technische Hochschule Zurich, 8093 Zurich, Switzerland

**Keywords:** SARS-CoV-2, solid-state NMR, structure, ORF7b, interactions

## Abstract

SARS-CoV-2 carries a sizeable number of proteins that are accessory to replication but may be essential for virus–host interactions and modulation of the host immune response. Here, we investigated the structure and interactions of the largely unknown ORF7b, a small membranous accessory membrane protein of SARS-CoV-2. We show that structural predictions indicate a transmembrane (TM) leucine zipper for ORF7b, and experimentally confirm the predominantly α-helical secondary structure within a phospholipid membrane mimetic by solid-state NMR. We also show that ORF7b forms heterogeneous higher-order multimers. We determined ORF7b interactions with cellular TM leucine zipper proteins using both biochemical and NMR approaches, providing evidence for ORF7b interaction with the TM domains of E-cadherin, as well as phospholamban. Our results place ORF7b as a hypothetical interferer in cellular processes that utilize leucine zipper motifs in transmembrane multimerization domains.

## Significance

The ORF7b accessory protein of SARS-CoV-2, a novel coronavirus responsible for the 2019 global pandemic, remains the least studied protein of the virus. While unraveling the molecular details of ORF7b would contribute to our overall understanding of coronaviruses, ORF7b has eluded standard structural approaches due to its small size, membrane-bound nature, and in particular its structural heterogeneity. Here, we gain insight into the structure and interactions of the protein using advanced biochemical and solid-state NMR methods. We expect that these protocols will be useful for future studies of similar proteins.

*Coronaviridae* are enveloped, single-stranded RNA viruses known to cause respiratory disease in animals and humans. One member of this family, Severe Acute Respiratory Syndrome Coronavirus 2, or SARS-CoV-2, spread rapidly and widely in late 2019, leading to the COVID-19 pandemic. COVID-19 disease is characterized by a wide range of symptoms, from mild respiratory problems to severe pneumonia, acute respiratory distress syndrome and, in the worst cases, death. In fact, the 2019 outbreak has created a global health crisis with significantly more deaths than the historically severe MERS-CoV and SARS-CoV outbreaks, while other strains are relatively harmless, such as those associated with the common cold. The emergence of most pathogenic strains has been associated with recent transmission of the virus from an animal reservoir, possibly from bats for SARS-CoV-2 (1) via other mammalian hosts, to humans (2).

*Coronaviridae* envelopes display spike proteins on their surface that help the virus enter host cells. In addition, the viral envelope contains the envelope (E) and matrix (M) proteins. The viral particles contain positive-sense, single-stranded RNA wrapped in nucleoproteins. The SARS-CoV-2 genome also encodes an extended set of nonstructural proteins, each with a specific function in hijacking the host's cellular machinery for replication and propagation (3). In total, the coronavirus genome contains 14 open reading frames that are predicted to encode 27 proteins, including four structural and nine accessory proteins (4, 5). These lesser-known but equally important accessory proteins play a fundamental role in viral replication, virulence, and evasion of the host immune response (6).

As their name suggests, accessory proteins are not required for the viral replication, but they may be important in mediating the host response to the virus, which can affect its pathogenicity and virulence (7), e.g., through interactions with host proteins (8). Accessory proteins can also be part of the viral particle (9, 10). It is these accessory proteins that are highly variable between viruses. In fact, while structural and nonstructural proteins are more than 90% conserved between SARS-CoV-2 and SARS-CoV and Bat-SL-CoVZXC21, the conservation of accessory proteins can be as low as 32% (11). The virulent strains MERS-CoV, SARS-CoV, and SARS-CoV-2 have a significant number of these proteins suggesting that they are important virulence factors capable of inducing complications not seen in less virulent coronavirus infections (7). Indeed, a substantial body of evidence has accumulated suggesting that accessory proteins make an important contribution to viral fitness. SARS-CoV-2 carries accessory proteins not previously observed in any human coronavirus (5). Elucidating the mechanisms by which these proteins function, or perturb the functions of host cells, may be the key to developing more effective treatments, vaccines, and antiviral strategies. Since the onset of the pandemic, three accessory protein structures have been solved: ORF3a (12), the ectodomain of ORF7a (13), and ORF8 (14) while the others could not be structurally characterized. In fact, the remaining accessory proteins, ORF3b, ORF6, and ORF7b, are small and membrane-interacting, making them difficult targets for structural biology.

Among these accessory proteins, ORF7b is one of the least studied. It has been reported that ORF7b plays an important role in modulating the IFN-interferon pathway (15) such as suppressing IFN-interferon production during early infection which facilitates evasion of host detection (16), or induction of cell apoptosis by promoting the expression of IFN-β, TNF-α and IL-6. In addition, it has been suggested that ORF7b may form a viroporin in its multimeric form (17). Additional functions are likely; it has recently been shown that among the SARS-CoV-2 proteins, ORF7b displays, together with N and ORF9b, the highest number of interacting proteins, and is involved in numerous interactions with proteins involved in glycosylation, neutrophil-mediated immunity, Golgi/ER transport, glucose metabolism, and transcription (18). E-Cadherin (E-cadTM) (Cadherin-1, CDH1) is one of the proteins shown to interact with ORF7b in this study (18).

ORF7b is a 43-residue protein of SARS-CoV-2 with approximately 80% sequence conservation to its SARS-CoV homolog. Interestingly, it shares more than 93% sequence homology with a bat coronavirus ORF7b protein (8) (*SI Appendix*, Fig. S1*A*). The ORF7b sequence clearly shows a long hydrophobic stretch, identified early on as a transmembrane domain (8), with a luminal N terminus and cytoplasmic C terminus. The transmembrane domain of ORF7b has been shown to be necessary for its retention in the Golgi complex (19). In vivo animal studies in transgenic mice SARS-CoV-2 mutants carrying complete deletions of ORF7b did not display significant differences in the pathogenesis (20). SARS-CoV ORF7b has also been shown to be integrated into viral particles, making it a structural component of the SARS-CoV virion (21).

We have previously reported the production of ORF7b by wheat-germ cell-free protein synthesis (WG-CFPS) in presence of detergent (22). Building on this, here we investigate the structure and interactions of the protein using biochemical approaches and solid-state NMR. Our experiments provide evidence that the protein forms higher-order multimers of α-helices. In addition, we show that ORF7b interacts in vitro with the TM domains of two central human cellular proteins, E-cadTM and phospholamban (PLN), which are involved in cell adhesion (23) and heart-beat regulation (24, 25), respectively.

## Results

### A Structural Model for ORF7b.

**Fig. 1.**
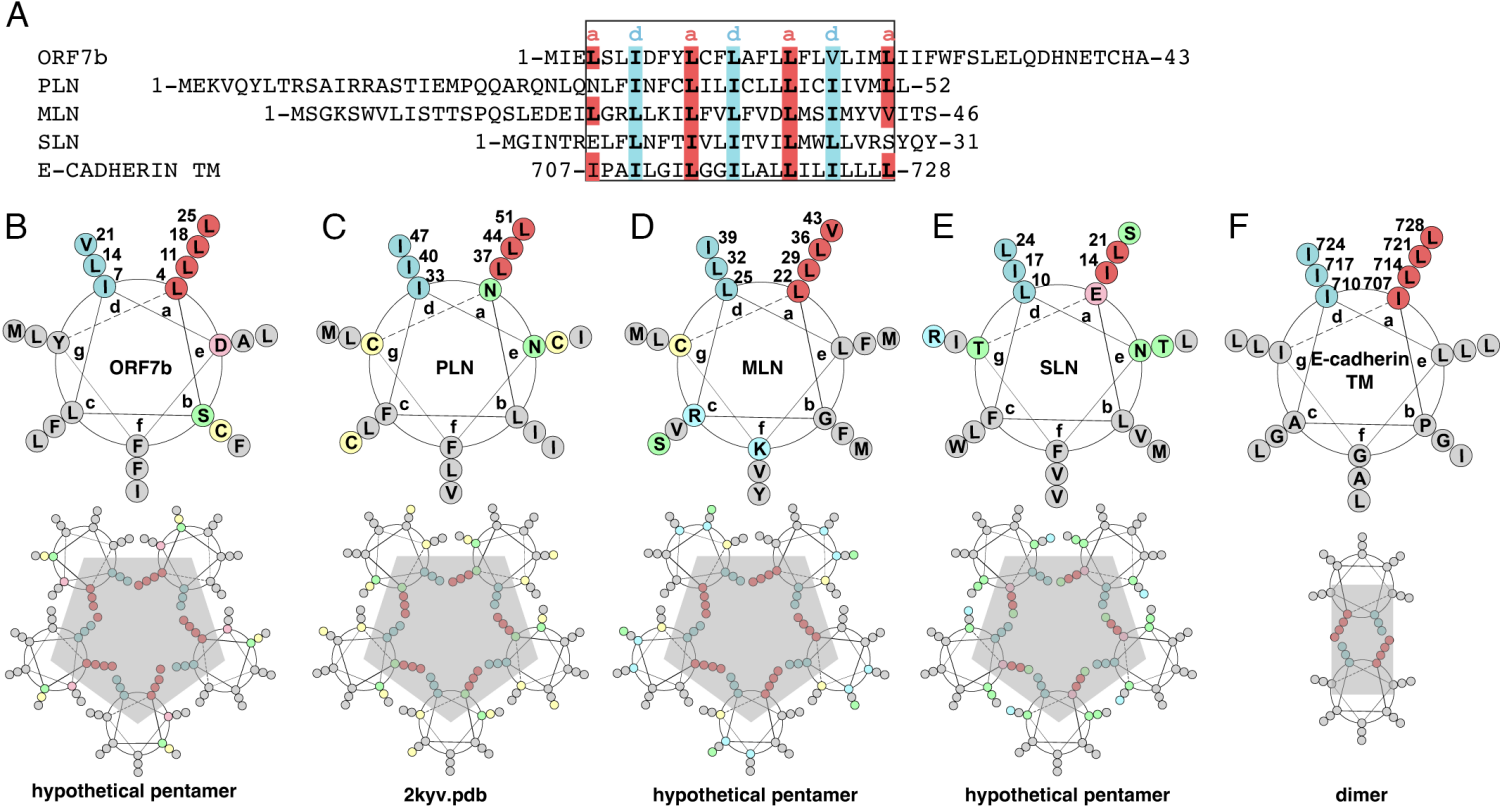
Leucine zipper motifs in ORF7b and cellular proteins. (*A*) Sequence alignment of ORF7b, PLN, MLN, SLN, and the transmembrane domain of E-cadTM, “a” and “d” positions of the helices, forming the interfacial leucine zipper, are highlighted in red and cyan, respectively. (*B*–*F*) Helical wheels of the proteins, with hydrophobic positions in gray, except for the a and d positions which are highlighted in red and blue, respectively. Polar residues are shown in green, acidic in pink, Cys in yellow, and basic in blue. The wheels were made based on the output of DrawCoil (https://grigoryanlab.org/drawcoil/). While the structure of PLN is experimental (25), the pentameric structures for ORF7b, MLN, and SLN are modeled based on PLN. The expected dimer structure model (23) is shown for the E-cadTM.

An analysis of the ORF7b sequence in a helical wheel plot illustrating the properties of alpha helices in proteins immediately leads to the identification of a canonical leucine zipper motif (26), as shown in Fig. 1 *A* and *B*. While this is a common interaction motif in soluble proteins, only few such sequences have been identified in membrane-bound proteins. The most prominent example is PLN, and by homology sarcolipin (SLN) (27) and myoregulin (MLN) (28). Another candidate is the TMD of E-cadTM, for which a dimeric Leu zipper motif has been described (23). Fig. 1*A* shows the sequences aligned to the Leu zipper motif as described in the helical wheels (Fig. 1 *B*–*F*). Starting from the PLN structure, which was experimentally determined to be a pentamer (25), one can also construct hypothetical multimeric models for ORF7b, MLN, and SLN, shown in the lower panels, displaying how the zipper could form the intermolecular interaction sites. For E-cadTM, Fig. 1*F* shows how Leu and Ile residues could interdigitate to form the TMD dimer (23).

**Fig. 2.**
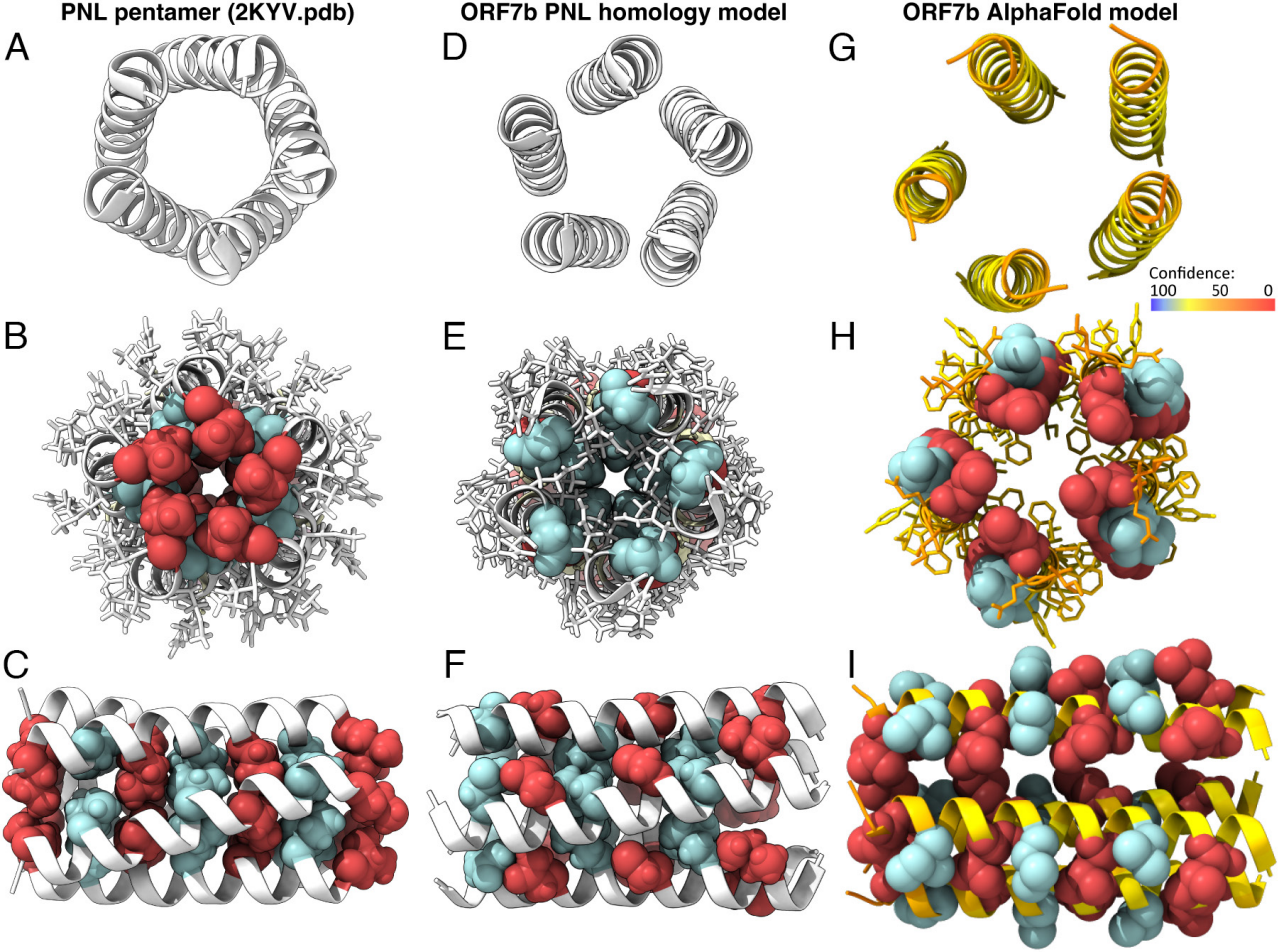
PLN 3D structure in comparison with ORF7b transmembrane leucine zipper structural models from PLN homology modeling and AlphaFold. (*A*–*C*) show the zipper portion of the transmembrane domain of the PLN pentamer structure [PDB 2KYV (25)]; (*D*–*F*) the corresponding portion of the ORF7b model created using intra- and intermolecular restraints (*SI Appendix*, Tables S1 and S2) measured on PLN and used in CYANA; and in (*G*–*I*) the model using AlphaFold. Panels *A*, *D*, and *G* show the top view of the coiled coil pentamer, and *B*, *E*, and *H* the same view with the zipper residues in spheres and the other residues in sticks. Panels *C*, *F*, and *I* show the zipper from the side, with sites a and d highlighted in red and blue, respectively (see also Fig. 1). The figure was generated using ChimeraX (32), with the protein backbone colors in panels G-I reflecting the reliability of the prediction as assessed within the AlphaFold pLDDT values, with dark blue (100), light blue (90), yellow (70), orange (50), to red (0). pLDDT > 90 indicate a model with high accuracy; 70 and 90 a generally good backbone prediction; 50 and 70 low confidence, and <50 not reliable.

To establish a molecular view of ORF7b, we used the structure of the PLN transmembrane helices [PDB 2KYV (25)], shown in Fig. 2 *A*–*C*, to generate a homology model using CYANA (29) and corresponding PLN distances as input (*SI Appendix*, Tables S1 and S2). Fig. 2 *D*–*F* show that a similar arrangement of the leucine zipper is sterically possible for a hypothetical ORF7b pentamer. However, the leucine zipper motif is not recognized as an interaction interface when AlphaFold (30) is used to predict the structure of the ORF7b multimer, as shown in Fig. 2 *G*–*I* using a pentamer as an example. Indeed, AlphaFold tends to position the Phe residues in the center. However, the low pLDDT values (an internal AlphaFold measure of model confidence), as reflected in the yellow-orange color coding, indicate that the prediction cannot be considered accurate (Fig. 2 *G*–*I*). It should be noted that this pattern also differs from the arrangement found in SARS-CoV-2 E (31), which in fact contains a similar FLAFxxF motif as ORF7b, where x is Leu in ORF7b and Val in E. In E, the side chains of the aromatic residues of this motif are oriented to the outside of the pentameric structure (31), as are the two Phe residues in PLN, and those in the ORF7b model built by homology to PLN.

To summarize, our analyses establish two different ORF7b multimeric models, one containing a Leu zipper motif in analogy to PLN, and the other one showing α-helical interfaces involving the opposite site of the helix, in particular Phe residues.

### ORF7b Forms Heterogeneous Assemblies.

**Fig. 3.**
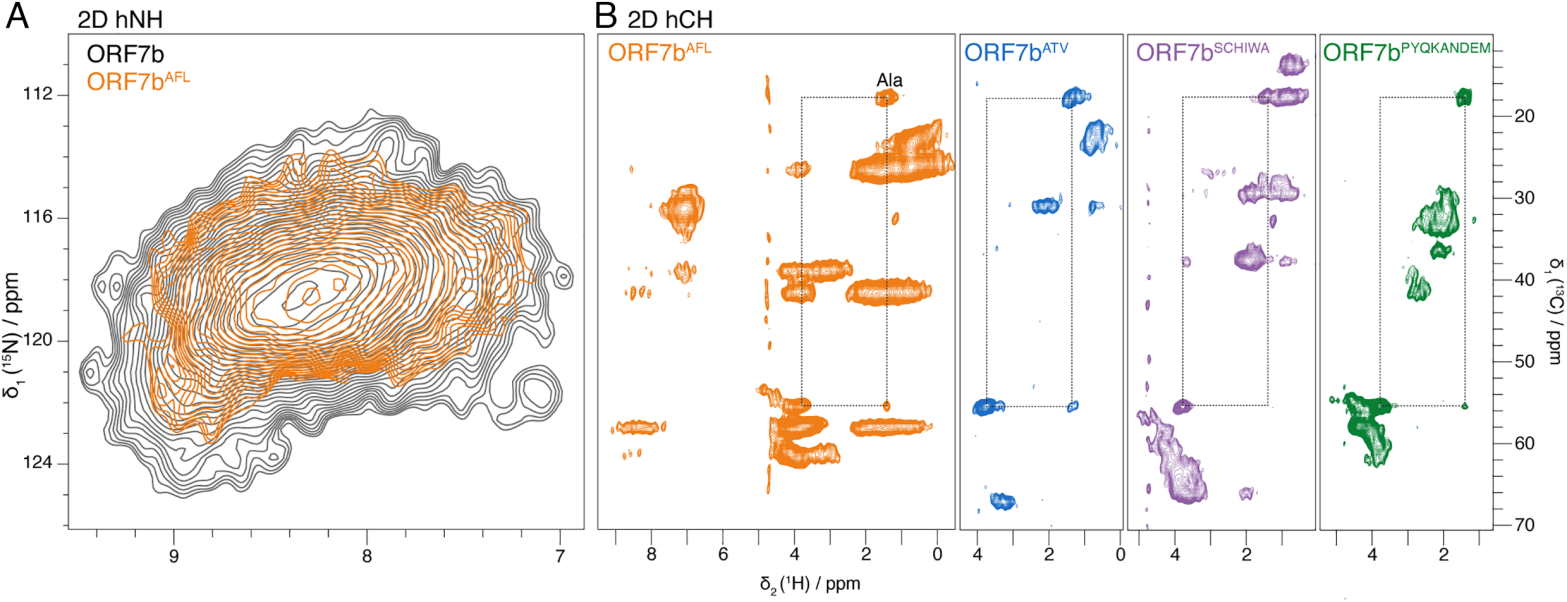
(*A*) 2D hNH spectra of ^2^H^13^C^15^N-ORF7b (gray) and ORF7b^AFL^ (orange) and (*B*) 2D hCH spectra of ORF7b^AFL^ (orange), ORF7b^ATV^ (blue), ORF7b^SCHIWA^ (purple), and ORF7b^PYQKANDEM^ (green). The Ala spin system is indicated by lines. All spectra were recorded at 100 kHz MAS frequency on a 850 MHz spectrometer. All proteins carry a Strep tag (WSHPQFEK), which was however not observed in the spectra, as indicated by the absence notably of E, K, H, P, and Q resonances.

We have previously shown that WG-CFPS can be used to produce ORF7b (22). For solid-state NMR samples, we have now reconstituted ORF7b at a lipid-to-protein ratio (LPR) of 0.5 (*SI Appendix*, Fig. S1 *C* and *D*) in an ERGIC lipid mixture [(33), *SI Appendix*, Table S3], chosen to mimic the lipid composition of the intermediate compartment between the ER and the Golgi which constitutes the main assembly site of coronaviruses (34). We first recorded a proton-detected 2D hNH spectrum at 100 kHz magic angle spinning (MAS) frequency of {^2^H,^13^C,^15^N} labeled ORF7b, shown in Fig. 3*A* in gray contours. Its unresolved features reveal that the individual ORF7b resonance lines must be considerably broader than in other proteins; see, e.g., refs. 35–37. Despite the limited resolution, we can see that the chemical shifts are clearly α-helical when comparing them to secondary-structure-specific chemical shifts (38), suggesting that the protein is not aggregated. To improve the spectral resolution, we prepared selectively labeled ORF7b^AFL^ (the superscript denotes {^1^H,^13^C,^15^N} labeled amino acids, while all others are {^2^H,^12^C,^14^N}). While the hNH spectrum is not improved (orange in Fig. 3*A*), the hCH spectrum (Fig. 3*B*) shows more resolution, as for example the separate resonance line for the Ala spin systems (*SI Appendix*, Fig. S2). This Ala resonance line is also observed in all the other selectively labeled samples we have prepared, which together cover all the remaining amino acids, plus Ala each time (Fig. 3*B*). We evaluated the proton line full width at half height (FWHH) of the Ala CH_3_ resonances in the hCH spectra, which we found to be 200 to 320 Hz (*SI Appendix*, Fig. S2). The upper limits of the carbon and nitrogen line widths, which are however sampling limited, can be estimated from the 3D hCNH spectra to be 300 to 440 Hz and 110 to 160 Hz, respectively (*SI Appendix*, Fig. S3). We have also evaluated whether a higher LPR would improve line widths. As seen in *SI Appendix*, Fig. S4 at the example of ORF7b^ATV^, the lines are only slightly narrower (by 35 Hz, 238 to 203 Hz) when going from LPR 0.5 to 4, and considering the significant loss in S/N, we stayed with LPR 0.5.

To further investigate the origin of the broad proton linewidths, we determined the bulk homogeneous relaxation times (T_2_′), the rotating-frame relaxation times (T_1ρ_) as well as the total linewidth (*SI Appendix*, Fig. S6 *A*–*C*). Individual relaxation curves are shown in *SI Appendix*, Fig. S5. Taken together, they characterize the coherent and incoherent contributions to the linewidths caused by the remaining dipolar interactions and molecular motion, respectively. In addition, there can be inhomogeneous contributions caused by sample inhomogeneities. The results are summarized in *SI Appendix*, Table S4. The ^N^H T_2_′ in deuterated ORF7b is 4.25 ms, which is comparable to the hepatitis C virus NS4B membrane protein (39). The relatively short relaxation times of ORF7b suggest possible contributions from incoherent line broadening caused by molecular dynamics. We next determined the proton inhomogeneous line widths, which mainly characterize the sample inhomogeneity (*SI Appendix*, Fig. S6 *D* and *E*). The resulting 79 to 210 Hz (*SI Appendix*, Table S4) show that inhomogeneity is a significant line-broadening mechanism in ORF7b. The rotating-frame relaxation time T_1ρ_ of ORF7b is close to 20 ms, which is on the order of that measured for hepatitis C virus NS5A (40) and apoptosis-associated speck-like protein (ASC) (41), but half that of the HBV capsid (37).

In conclusion, lipid-reconstituted ORF7b shows broader lines and faster relaxation than other protein samples studied by solid-state NMR. We identified mainly heterogeneous, but likely also incoherent, contributions as the cause. In order to reduce resonance overlap, we therefore used the selectively labeled ORF7b samples shown in Fig. 3*B* for further analysis.

### NMR-Derived Boundaries and Secondary Structure of ORF7b.

**Fig. 4.**
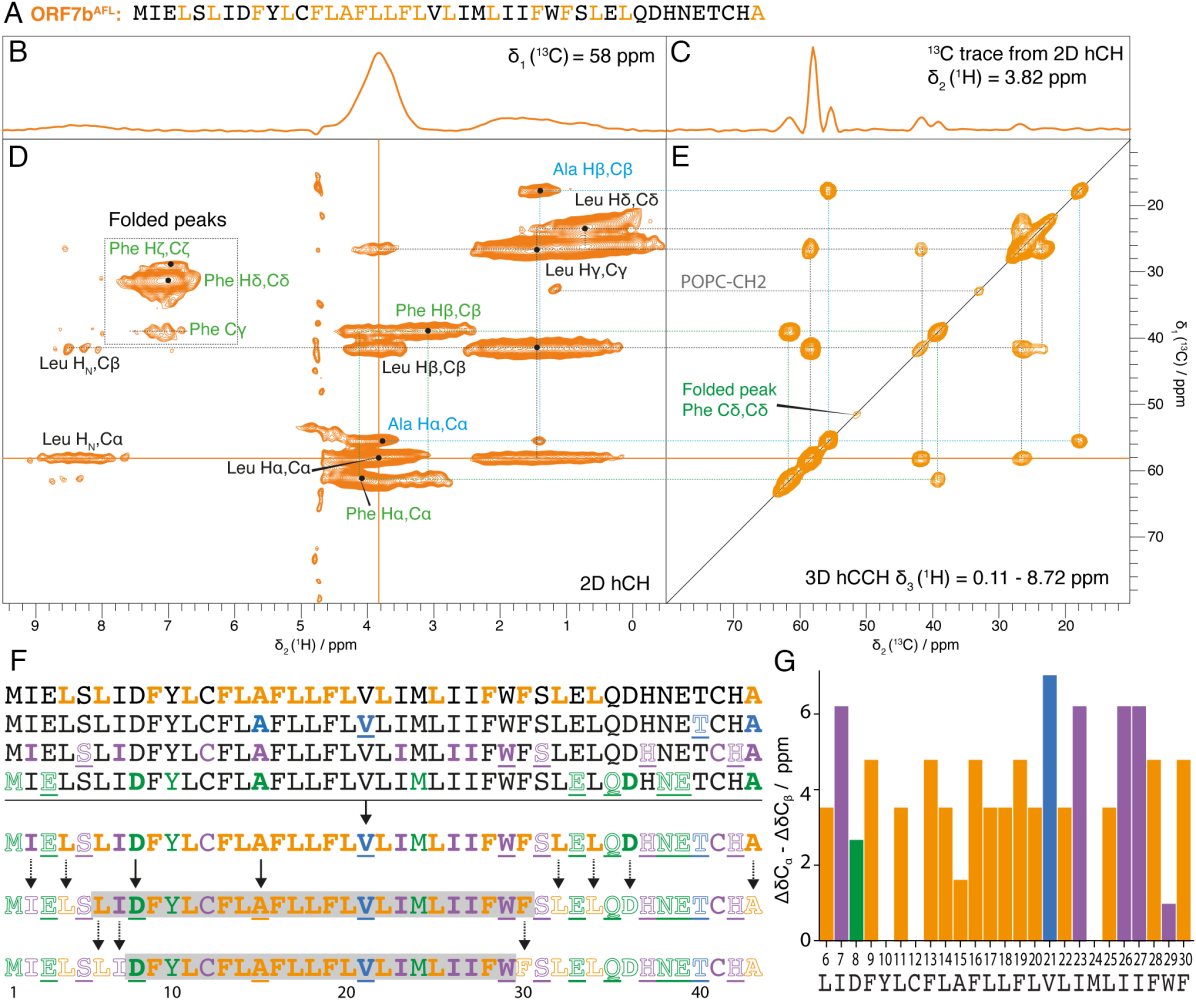
Secondary structure of the rigid ORF7b domain. (*A*) Sequence and amino-acid labeling of ORF7b^AFL^. (*B*–*D*) 2D hCH spectrum of ORF7b^AFL^ with two 1D ^1^H and ^13^C extracts. Assignments of the observed signals to the different amino acids are given, in blue for Ala, in green for Phe, and in black for Leu. (*E*) 3D hCCH spectrum, all planes between 0.11 and 8.72 ppm are shown. (*F*) Summary of the observed and assigned ORF7b amino acids, and conclusions drawn with respect to the extent of the rigid portions (see main text for details). (*G*) Secondary chemical shifts (SCS) which indicate α-helical secondary structure when four or more residues in a row are positive, and β-strands when three or more residues in a row show negative values (not observed here). The SCS values are calculated based on the averaged chemical-shift values (44). The ORF7b sequence between Leu6 and Phe30 is shown, with the same value for all amino acids of the same type as they cannot be distinguished experimentally.

AlphaFold predicts an uninterrupted α-helical structure for residues 2–42 of ORF7b (*SI Appendix*, Fig. S1*B*), whereas a PLN homology model positions only residues 4–25 as a helical TM portion. We therefore set out to experimentally determine the C^α^ and C^β^ NMR chemical shifts that can be translated into secondary structures (42). For this purpose, we recorded 2D hCH and 3D hCCH TOBSY (43) spectra of ORF7b^AFL^, ORF7b^ATV^, ORF7b^SCHIWA^, ORF7b^PYQKANDEM^ (Fig. 4 *A*–*D* and *SI Appendix*, Figs. S8–S10). We also recorded INEPT spectra (*SI Appendix*, Figs. S7–S10), but since they could only be tentatively assigned, we did not use them further in our analysis.

The 3D hCCH spectra were sufficiently resolved to assign resonances to amino-acid types based on the typical chemical shifts and spin systems, as shown in Fig. 4*E* for ORF7b^AFL^ and *SI Appendix*, Figs. S8–S10 for the other samples. The chemical shifts are summarized in *SI Appendix*, Table S5. Spin systems can be identified for several amino acids, indicating that at least one of the occurrences must be part of the rigid portion of ORF7b. In all samples, single, albeit broadened, signal sets are observed for a given amino acid, indicating that the variation in dihedral angles between residues of the same amino acid type does not result in chemical-shift differences that exceed the ^13^C linewidth as caused by the inhomogeneity of the sample. The assignments are therefore not site-specific but only amino-acid-specific, e.g., the Ala peak can in principle originate from Ala15 or Ala43 or both. Exceptions are Val, Tyr, Trp, Thr, Asn, and Gln, which occur only once in the sequence.

To derive the rigid fraction of the protein, we first cataloged the residues observed in the 3D hCCH spectra and marked them in bold on the sequences in Fig. 4*F*. The unobserved residues are shown in outline letters. Rows 1 through 4 represent the four isotopically labeled samples. Next, we determined which observations were unambiguous. This is the case for observed amino acids with a single occurrence in the sequence, as well as for unobserved residues. A summary of these direct observations is given in the fifth row. Note that Cys, Tyr, and Met residues do not fall into a clear category because they do not show correlation signals in 3D, but signals with corresponding chemical shifts are present in 2D spectra (Cys) or on the 3D hCCH diagonal (Met, Tyr). Next, we consider that the residues observed in the spectra but occurring multiple times in the sequence may not all be rigid. This led us to remove the Ala, Asp, Ile, and Leu residues that are interspersed among the unobserved residues, as indicated by the dashed arrows. As a result, the presence of Asp8 and Ala15 in the rigid part is now obligatory (black arrows). The gray box indicates the maximum plausible extent of the rigid region, and the next row indicates the minimum. We then analyzed the SCS of these residues, as shown in Fig. 4*G* and *SI Appendix*, Table S6. It can be seen that all rigid amino acids have α-helical SCS.

Taken together, our analyses reveal a rigid α-helical segment encompassing residues 8–29, which, based on its high hydrophobicity, can be identified as the ORF7b TMD. This segment is significantly shorter than the helix predicted by AlphaFold, and slightly shifted when compared to the PLN-based model.

### ORF7b Forms Higher-Order Multimers.

**Fig. 5.**
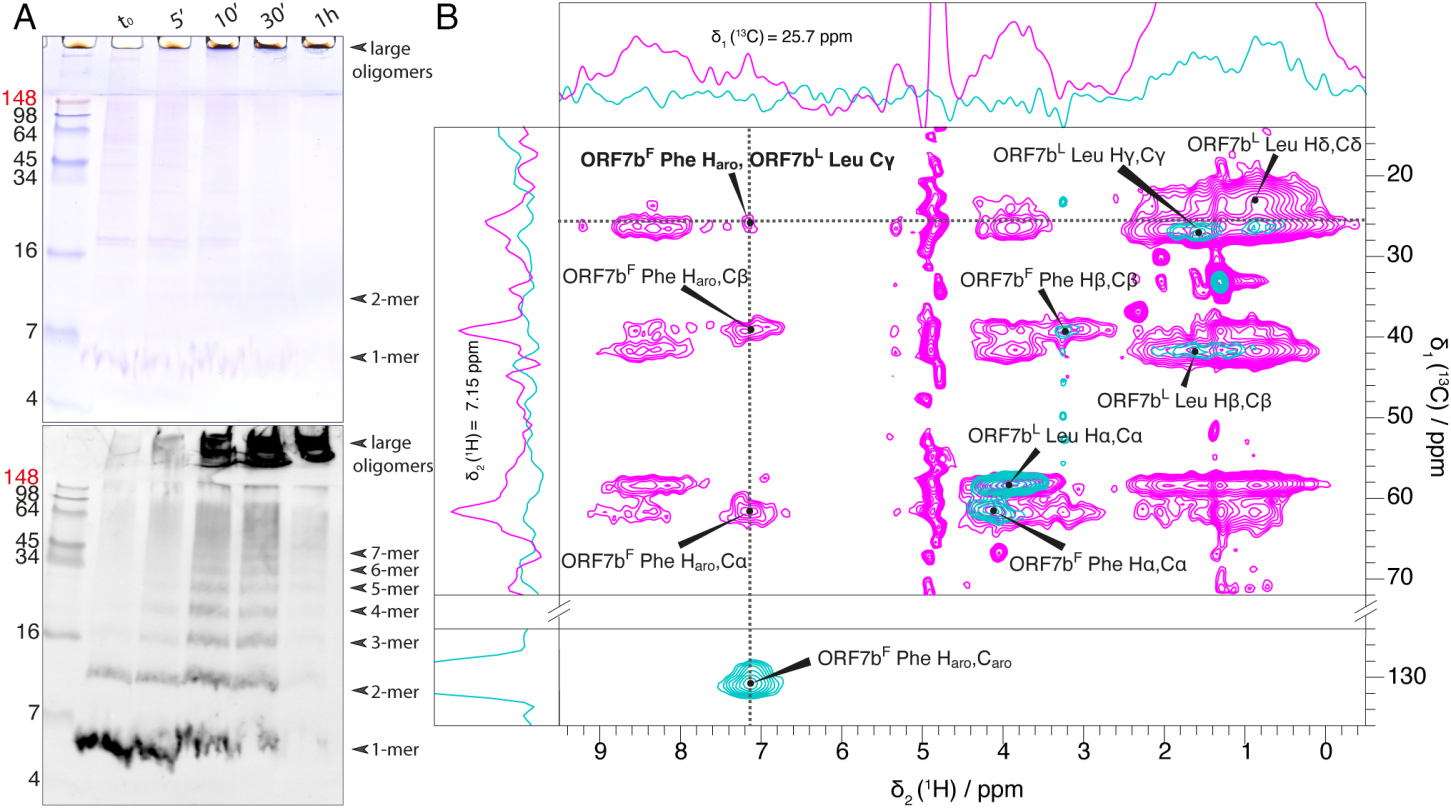
ORF7b forms multimers. (*A*) Cross linking experiments show that with increasing incubation time in presence of DMTMM, a zero-length crosslinking agent, the amount of ORF7b monomer (1-mer) reconstituted in lipids with an LPR of 20 decreases. The poor quality of the bands is due to the high amounts of lipids present. For crosslinking in detergent, see *SI Appendix*, Fig. S12. (*B*) Intermolecular polarization transfer between ORF7b molecules. 2D hChH-MIRROR spectra of mixed labeled ORF7b^L:F^ recorded at 1.2 GHz at 23 °C with 50 ms mixing time (NS = 1,280) in magenta, overlaid for reference on 2D hCH ORF7b^L:F^ spectra (NS = 64) in teal. The frequency of the MIRROR irradiation was set to the resonance frequency difference of ^1^H_γ_ of Leu and aromatic ^1^H of the Phe residues (6.6 kHz). The cross peak showing the intermolecular correlation is labeled in bold type. 1D traces are shown for the frequencies labeled with dotted lines.

To validate the possible formation of multimers by ORF7b, we performed a chemical cross-linking experiment using 4-(4,6-dimethoxy-1,3,5-triazin-2-yl)-4-methylmorpholinium chloride (DMTMM) on both lipid-reconstituted ORF7b (Fig. 5*A*) and the protein in detergent (*SI Appendix*, Fig. S12). DMTMM cross links carboxylic acids to primary amines, as occurring in Lys or Arg (45). In ORF7b, Lys is present in the Strep tag. As shown in Fig. 5*A* on the Western blot, evidence of ORF7b multimers is observed, with dimers already present at t = 0. Subsequently, higher-order multimers become apparent, although without a specific preference for a specific size. Notably, we clearly identified up to 7-mers, but higher aggregates are also observed, including in the gel stackings.

To confirm the homologous ORF7b interactions by NMR, we prepared a sample from a 1:1 mixture of Leu- and Phe labeled ORF7b, which we name ORF7b^L:F^. In addition to being very abundant, Leu and Phe also present the important advantage that their signals in the aromatic and aliphatic ^13^C/^1^H regions show excellent spectral separation. To detect intermolecular contacts, we recorded a hChH correlation spectrum on ORF7b^L:F^ which is shown in pink in Fig. 5*B* (for the pulse sequence, see *SI Appendix*, Fig. S11). To clearly identify intermolecular cross peaks, we compared this spectrum with a hCH spectrum, which shows only intraresidue correlations (in teal in Fig. 5*B*). A distinct Phe-Leu correlation peak is observed in the hChH spectrum, the traces for which are shown at the *Top* and *Left* in Fig. 5*B*. To ensure that no alternative mechanism could lead to this correlation peak, we performed several controls to exclude that lipid signals could be the origin of the correlations signal (*SI Appendix*, Fig. S13 *A*–*D*). For this purpose, we used deuterated lipids (*SI Appendix*, Fig. S13*A*), ORF7b with a Phe label but no Leu label (*SI Appendix*, Fig. S13*B*), and a lipid sample in the absence of protein (*SI Appendix*, Fig. S13*C*). All controls were negative. The detection of this peak provides thus clear evidence of an intermolecular close contact between Leu and Phe residues. Measurement of NMR intermolecular restraints using rather Leu/Ile than Leu/Phe residues would have allowed to probe in addition whether intermolecular contacts involve the residues of a hypothetical zipper structure. However, it would have been nearly impossible to resolve intermolecular cross peaks between these residue types because their aliphatic proton resonances show extensive overlap.

To summarize, we conclude from the biochemical and NMR experiments that ORF7b forms oligomers, via homologous interactions involving at least one Leu/Phe residue pair.

### ORF7b Interacts with E-cadTM and PLN.

**Fig. 6.**
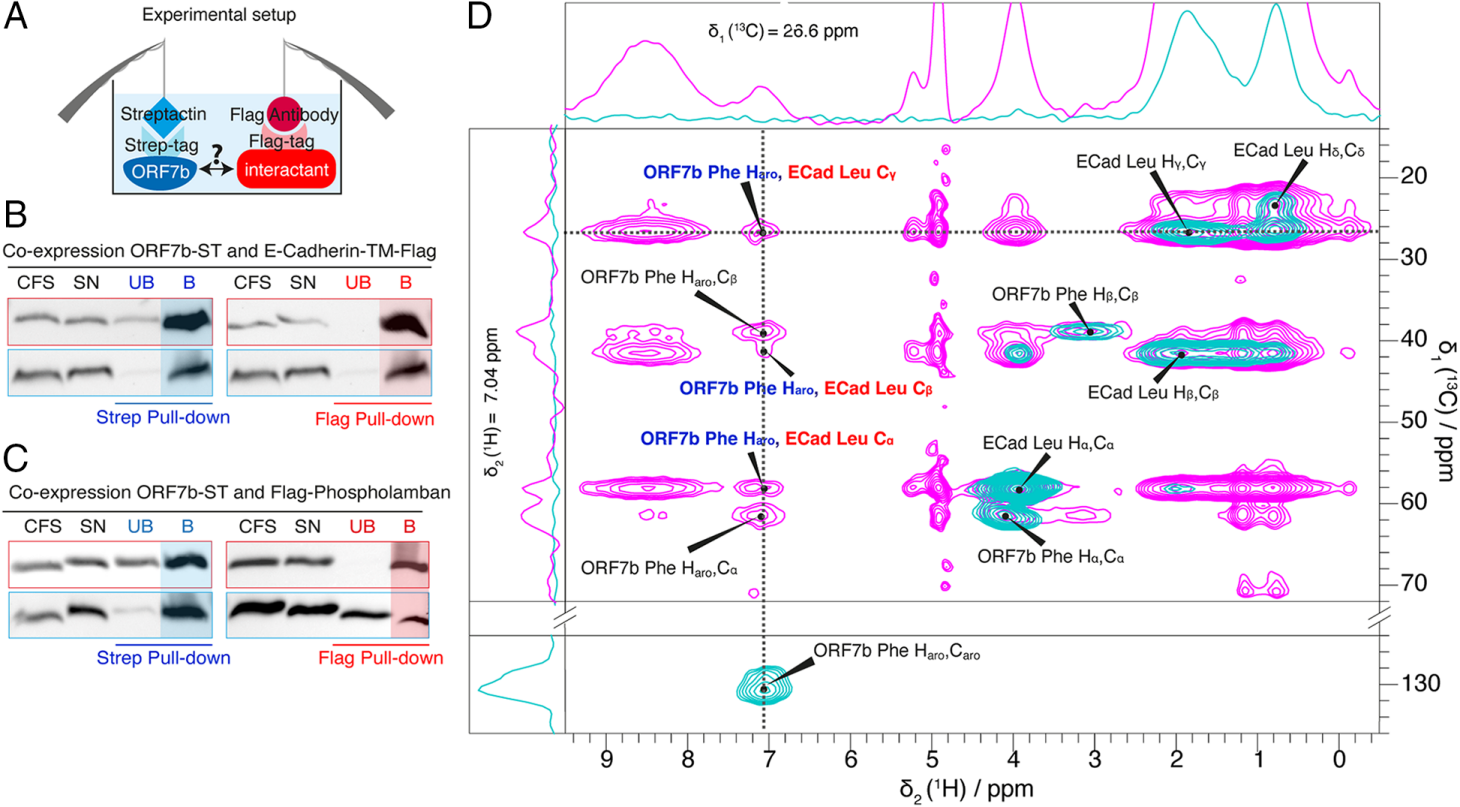
Interactions between ORF7b and E-cadTM/PLN. (*A*) Pull-down experimental setup. The soluble fraction (SN) of the cell-free synthesis (CFS) reaction was incubated with Strep-tactin or with Flag-antibody beads. Interactions between the proteins lead to their cocapture. Indeed, the western blots show that ORF7b and (*B*) E-cadTM or (*C*) PLN are cocaptured in the bead fraction. Very little protein is detected in the UB besides a small band for ORF7b in the Flag pull-down (*C*, *Right* panel). In (*D*), the 2D hChH reverse-MIRROR spectrum of the ORF7b^F^:E-cadTM^L^ sample is shown (recorded at a field strength of 1.2 GHz at 19 °C with 50 ms mixing time (NS = 1,280) in magenta superimposed on the 2D hCH spectrum for reference (NS = 64) in cyan). The frequency of the MIRROR irradiation was set to the resonance frequency difference of ^1^H_γ_ of Leu and aromatic ^1^H of the Phe residues (6.6 kHz). The intermolecular cross signals are indicated with red/blue labels, while the intramolecular cross peaks are labeled in black. 1D traces are shown for the frequencies labeled with dotted lines.

To experimentally assess whether ORF7b actually interacts with the Leu-zipper proteins E-cadTM and PLN, we first coexpressed in the cell-free reaction each of the two proteins with ORF7b, followed by affinity capture on magnetic beads. While ORF7b carried a Strep tag, a Flag tag was used on the interacting protein. Both Strep-Tactin and Flag antibody magnetic beads were used for the pull-down assay (Fig. 6*A*) to detect interactions from both sides. As shown in Fig. 6*B*, cocapture of ORF7b and E-cadTM was observed using each types of beads, clearly demonstrating an interaction between the two proteins in the cell-free reaction. In the control (absence of ORF7b, *SI Appendix*, Fig. S14), E-cadTM is predominantly detected in the unbound fraction (UB). Similarly, the interaction between PLN and ORF7b was also confirmed by analyzing the bead fractions, where the proteins are cocaptured, both in the Strep or Flag pull-down (Fig. 6*C*). However, for PLN, a significant amount of interactants was not bound to the beads (~33% in the Strep pull-down and 50% of ORF7b in the Flag pull-down), indicating either a lower affinity between PLN and ORF7b when compared to the E-cadTM/ORF7b pair, or a higher stoichiometric ratio in the latter reaction.

We subsequently set out to observe the formation of the stronger E-cadTM/ORF7b interaction by NMR. The spectrum of E-cadTM is shown in *SI Appendix*, Fig. S15. To detect contacts between the two proteins at the atomic level, we prepared a sample containing a mixture of purified, selectively labeled E-cadTM^L^ and ORF7b^F^ in a molar ratio of 1:1 and recorded hChH correlation spectra analogous to those in Fig. 5*B*. Fig. 6*D* shows the spectrum in pink, with superimposed the hCH spectrum in teal. Correlation peaks between Leu (E-cadTM) and Phe (ORF7b) resonances can clearly be detected and demonstrate the presence of ORF7b/E-cadTM heteromultimers.

We thus conclude from our data that ORF7b interacts with the cellular membrane-spanning proteins E-cadTM and PLN.

## Discussion

For the SARS-CoV-2 accessory protein ORF7b in a lipid environment, we have determined that the rigid region of the protein spans minimally the range Asp8-Trp29, and maximally Leu6-Phe30. This rigid core shows exclusively α-helical secondary structure and corresponds, as supported by its near-exclusive hydrophobicity, to the OFR7b transmembrane helix. We have established that ORF7b forms homologous interactions through multimer formation, and also that multimers are formed in an inhomogeneous manner. We have importantly shown that ORF7b forms heteromultimers with two cellular proteins, E-cadTM, and PLN, that both display leucine zippers in their TMDs.

**Fig. 7.**
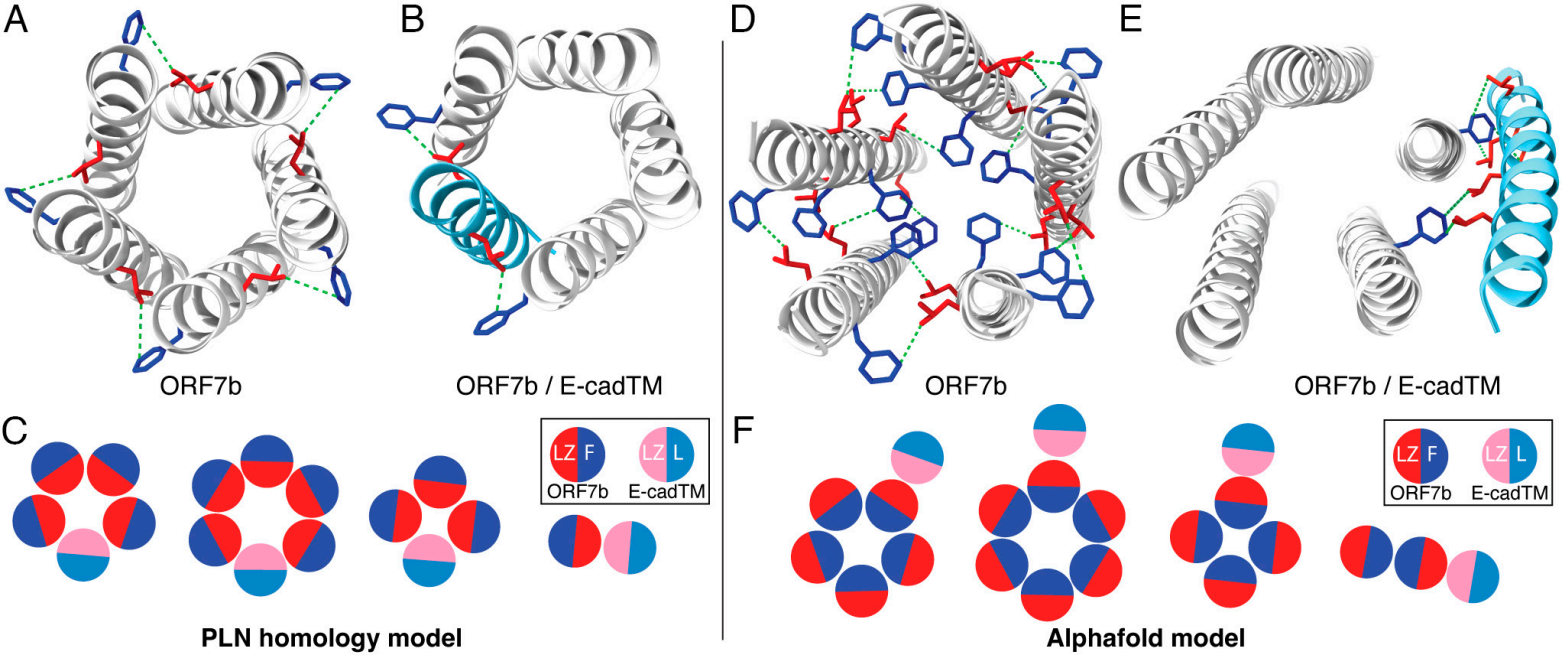
Compatibility of the ORF7b models with NMR data. (*A*) Distances smaller than 5 Å (green lines) between Leu and Phe protons on the pentameric PLN-based ORF7b homology model; (*B*) distances between ORF7b^F^ and E-cadTM^L^ residues on the pentameric ORF7b with E-cadTM insertion via Leu zipper interactions. (*C*) Sketch of the possible arrangement of ORF7b and E-cadTM, with red parts representing the ORF7b Leu zipper (LZ) motif, dark blue the Phe-rich side of ORF7b (F), pink the E-cadTM leucine zipper (LZ), and light blue the opposite leucine-rich side (L). (*D*–*F*) equivalent representations for the AlphaFold model.

NMR shows that the oligomers form contacts between Phe and Leu residues, both of which are abundant in ORF7b. Fig. 7 *A* and *D* show the ORF7b pentamer models created on one hand by homology modeling of the PLN structure (25) and on the other hand using AlphaFold (30). Only side chains for Phe and Leu residues which have protons that are closer in space than 5 Å, as indicated by green broken lines, are displayed. It can be seen that in both models Leu and Phe are close enough that NMR direct polarization transfer peaks can be expected, with the difference that they are more abundant in the AlphaFold model. Thus, we can say with confidence that the ORF7b monomers interact to form multimeric assemblies and that both possible interaction modes shown in Fig. 7 *A* and *D* are compatible with our data.

We also showed that ORF7b interacts with both PLN and E-cadTM. The interaction was more specifically probed by NMR between ORF7b and E-cadTM, which revealed intermolecular correlations between the abundant Phe and Leu residues labeled on ORF7b^F^ and E-cadTM^L^, respectively. Fig. 7 *B* and *E* shows how the two different ORF7b models could interact with E-cadTM. In the PLN homology model, the multimer is formed by the leucine zipper motif, and E-cadTM could replace an ORF7b monomer, resulting in a heteromultimer, as shown at the example of a pentamer in Fig. 7*B*. The situation is different for the AlphaFold model, where the leucine zipper residues are pointing to the outside, and an interaction with E-cadTM via the leucine zipper places the E-cadTM next to the ORF7b multimer (Fig. 7*E*). We built a model for this situation starting from a canonical leucine zipper coiled-coil [1p9i (46)] motif to position E-cadTM with respect to ORF7b via a Leu zipper motif. Distances <5 Å between Phe and Leu residues are present in both models, meaning that our data are again compatible with both.

We suspect that the heterogeneous line widths result from the fact that not a single interface exists between the proteins, i.e. that not always exactly the same residues interact with the same geometries as for example typically observed in transmembrane channels, but that interactions can take place via the highly hydrophobic monomer surfaces in multiple ways. Fig. 7 *C* and *F* summarizes these findings by sketching further possible arrangements, taking into account that other multimeric forms are possible, and that their coexistence is supported by the observation of broad and clearly non-Gaussian or Lorentzian, inhomogeneous ^1^H line width. Still, since there is no specific relationship describing precisely how linewidth correlates with heterogeneity, a quantitative characterization cannot be derived from our data.

It is interesting to note that ORF7b shows an exceptionally high level of transcript abundance in spite of low mRNA levels (47). One could speculate that such a hydrophobic TM protein, carrying in addition a leucine zipper motif used also by cellular proteins, if expressed at high copy numbers in cells, has the potential to interfere with correct targeting and recycling (48) of cellular proteins and lead to disruption of cellular processes involving these (or other TM) proteins. As shown by the ORF7b interactions revealed in our work, this has the potential to ultimately result in disturbances in cell adhesion (E-cad) and heart-rate regulation (PLN). Of note, one of the most common symptoms among the COVID-19 patients, i.e., loss of smell, is caused by disruption of cell adhesion in the olfactory epithelium sustentacular cells (49).

To verify the proposed interactions, we also searched for a virus variant lacking ORF7b and found one involving a mutation in the ORF7a stop codon (B.1.616) (50). This variant was detected in an outbreak of nosocomial cases of COVID-19. All severe cases caused by this variant had high-risk factors (age and/or comorbidities). This variant also had the peculiarity of not being detected in nasopharyngeal samples, which was found to be due to a viral load below the upper respiratory tract detection limit. The variant has not been observed beyond this outbreak, which may also be due to its poor detection. Thus, due to the small number of cases combined with the high comorbidity factors, it is very difficult to clarify the increased or decreased severity of this variant (50).

## Conclusion

We here show that ORF7b comprises an α-helical transmembrane domain and C-terminally dynamic residues, and that it assembles to form heterogeneous multimers. Our study importantly shows that ORF7b interacts with E-cadTM and PLN, two cellular proteins carrying a leucine-zipper motif. Based on our results, and the previous finding that ORF7b is expressed in high copy numbers in the cell, we speculate that ORF7b could impair the function of important host proteins, notably resulting in compromised cell adhesion and heartbeat regulation.

## Materials and Methods

### Model Building.

The model of ORF7b (residues 3–25) was built in homology to PLN (PDB 2KYV) (25) using CYANA (version 3.98.13) (29). The input for model building was the 44 (ϕ,ψ) torsion angle restraints (per monomer, identical for all monomers) set to the average values of the PLN leucine zipper structure, as well as 5 Cα–Cα and 5 Cβ–Cβ intramonomer distance restraints representing the i → i + 7 contacts for the zipper residues 4L, 7L, 11L, 14L, 18L, 21V, and 25L (*SI Appendix*, Table S1) which were set to the corresponding values in the PLN structure (pdb 2KYV) ± 0.25 Å. Thirty-three intermonomer restraint between the same resides on different monomers were used to restrain the pentamer; see *SI Appendix*, Table S2. C_5_ symmetry was implemented for the structural model. Flexible linkers between the monomers were inserted into the sequence for the CYANA calculation. The 10 best structures out of the structure bundle of 100 structures showed neither violations of distance or torsion-angle restraint, nor van-der Waals. The backbone RMSD of the model-bundle is 0.15 ± 0.03 Å.

### AlphaFold Predictions.

An implementation of AlphaFold2.1 (30) software on a local server was used for predictions. Amino-acid sequences were entered and predictions were run in the multimer mode. Per-residue estimates of the confidence of the models are given on a scale from 0 to 100 in the individual PDB files of the predictions (pLDDT).

### Plasmid.

The sequence of the full-length ORF7b from SARS-CoV-2 (GeneBank Accession No. 43740574) was synthesized and cloned into the pEU-E01-MCS vector (CellFree Sciences, Japan) with a SA linker and a Strep-tag II (SAWSHPQFEK) at the C terminus. The resulting plasmid was amplified in chemically competent *Escherichia coli* TOP10 cells (Life Technologies). DNA was isolated using a NucleoBond Xtra Maxi kit (Macherey-Nagel, France), and was further purified by a phenol/chloroform extraction.

Based on the full-length sequence of E-cadTM (GenID 999), the transmembrane domain sequence was synthesized to obtain the fragment between residues E641 and R673, which was cloned into the pEU-E01-MCS vector with either an SA linker and a Strep-tag or a GGG linker and a Flag-tag at the C terminus. For PLN, the full-length protein sequence (GeneID 5350) was cloned into the pEU-E01-MCS vector with a Flag-tag and a GGG linker (DYKDDDDKGGG) at the N terminus, just after the start methionine.

### WG-CFPS.

Cell-free protein synthesis was performed using homemade wheat germ extracts, as described previously (51–53). Briefly, transcription and translation steps were performed separately. Transcription was performed for 6 h at 37 °C in transcription buffer containing RNAsin (1 U/μL), SP6 polymerase (1 U/μL) (CellFree Sciences), 10 mM rNTP mix (Promega) and 0.1 μg/μL of plasmid DNA. Translation was performed for 18 h at 22 °C without shaking using the bilayer method. Protein production for further purification and lipid reconstitution was performed in 8 wells of 6-well plates. Translation reactions consisted of 0.5 mL of translation mix in the bottom layer, overlaid with 5.5 mL of feeding buffer in one well. The translation mix contained 0.25 mL of the transcription reaction, 0.25 mL wheat germ extract, 40 ng/mL creatine kinase, 0.1% (w/v) MNG-3 (Maltose Neopentyl Glycol-3, Anatrace), and 6 mM amino acid mixture (average concentration of each amino acid 0.3 mM). The latter was adjusted to the sequence composition of the protein for an optimal yield and to the desired labeling scheme for NMR (for a list of selectively labeled samples, see *SI Appendix*, Table S8). The feeding buffer consisted of 1x SUB-AMIX buffer (CellFree Sciences Japan), 0.1% MNG-3, and 6 mM amino acid mixture.

### SDS-PAGE and Western Blot Analysis.

SDS-PAGE and Western blot analysis were performed as described in ref. 54. Protein detection on the blots was performed with an antibody against the Strep-tag II (StrepMAB-Classic, IBA Lifesciences, Germany). Expression was assessed on 15% Coomassie blue-stained Tricine SDS-PAGE gels. Samples were resuspended in loading buffer containing 62.5 mM Tris-HCl pH 6.8, 2% SDS (w/v), 5% β-mercaptoethanol (v/v), 0.01% bromophenol blue (w/v), and 10% glycerol (v/v). For samples where sucrose gradients were used, no glycerol was added in the loading buffer. For each sample, 10 μL were loaded onto SDS-PAGE gels. Western-blot analyses were performed out by protein transfer to a nitrocellulose membrane using an iBlot® gel transfer device (Life Technologies). The nitrocellulose membrane was blocked with 5% nonfat milk powder in PBS-T buffer (12 mM sodium phosphate pH 7.4, 137 mM NaCl, 2.7 mM KCl, 0.05% Tween® 20). After blocking, the membrane was incubated with StrepMAB-Classic primary antibody (IBA Lifesciences, Germany) or the mouse anti-FLAG M2 antibody (Sigma-Aldrich) for 1.5 h at RT, followed by anti-mouse IgG HRP-conjugated secondary antibody (Promega, France) for 1 h at RT. The blots were then detected with ECL Prime Western Blotting Detection Reagent (GE Healthcare, France).

### Purification of ORF7b by Affinity Chromatography.

After cell-free protein synthesis, the total cell-free reaction of 45 mL was incubated with benzonase, and 0.1% (w/v) DDM (N-dodecyl-beta-maltoside) to exchange from MNG-3 to DDM detergent, for 30 min at room temperature on a rolling wheel. The total reaction was then centrifuged at 20,000×*g* and 4 °C for 30 min, and the supernatant was applied to a 1-mL Strep-Tactin column (IBA Lifesciences). Purification was performed according to the manufacturer’s instructions, with all buffers containing 0.1% DDM. ORF7b was eluted in 100 mM Tris-HCl pH 8, 150 mM NaCl, 1 mM EDTA, 0.1% DDM, and 5 mM desthiobiotin.

### Reconstitution of ORF7b in Lipids.

The ERGIC lipids were prepared by mixing 1-palmitoyl-2-oleoyl-glycero-3-phosphocholine (POPC—Avanti 850457), 1-palmitoyl-2-oleoyl-sn-glycero-3-phosphoethanolamine (POPE—Avanti 850757), L-α-phosphatidylinositol (liver PI—Avanti 840042), 1-palmitoyl-2-oleoyl-sn-glycero-3-phospho-L-serine (POPS—Avanti 840034), and cholesterol (Sigma-Aldrich C3045) in the ratio given in *SI Appendix*, Table S3. The lipid mixture was solubilized in Triton X-100 10-fold molar excess to the lipids. Purified ORF7b in DDM micelles was then mixed with ERGIC lipids and incubated at 4 °C for 1 h. Detergent was removed by stepwise addition (25 steps, every 15 min) of β-methyl-cyclodextrin (50 %) to a final concentration of 8% (w/v) and the solution was incubated at 4 °C for 72 h. For ORF7b^FC^ and the mixture ORF7b^FL^, detergents were removed by adding SM-2 Bio-Beads (Bio-rad) at 200 mg per ml of solution. The mixture was then incubated at RT followed by an O/N incubation at 4 °C with fresh beads. The resulting LPR was 0.5 (w/w). Proteoliposomes were isolated from empty liposomes or aggregated protein on a sucrose density gradient. ORF7b proteoliposomes were mainly found in the 30 to 50% sucrose layer (*SI Appendix*, Fig. S1*C*). These fractions were pooled and the resulting sediment (*SI Appendix*, Fig. S1*D*) was filled into 0.7 mm NMR rotors.

### Coexpression of Two Proteins Using the Cell-Free System.

Coexpression was performed in a small-scale CFS reaction according to the protocols described above. The cell-free transcribed mRNAs of ORF7b-ST and E-cadTM-Flag or Flag-PLN were added together in equimolar amounts to the cell-free translation reaction, together with 0.1% (w/v) MNG3 in order to obtain soluble protein. After the translation step, the CFS reaction was centrifuged at 20,000×*g* for 30 min at 4 °C. The pellet was discarded and the supernatant was incubated with either Strep-Tactin (5 µg beads, for 1 h at 4 °C) or Flag magnetic beads (10 µg, for 3 h at 4 °C). After the incubation time, the beads were separated from the unbound/flow-through fraction using the Promega magnetic stand.

### Cross-Linking Experiments.

The purified ORF7b in lipids (LPR 20) and DDM micelles was exchanged into crosslinking buffer (50 mM HEPES pH 7.4, 100 mM NaCl, and 12 mM TCEP). DMTMM, a crosslinking agent that forms covalent bonds between the primary amines of Lys and Glu/Asp residues in ORF7b, was added to a final concentration of 7 mM and incubated at 37 °C. A 20 µL sample was collected for analysis at 5, 10, 30, 60, and 120 min.

### Solid-State NMR.

Solid-state NMR experiments were recorded using a Bruker AVANCE III wide-bore spectrometer operating at a ^1^H resonance frequency of 850 MHz and a Bruker AVANCE NEO standard-bore spectrometer operating at a ^1^H resonance frequency of 1.2 GHz, and internally referenced to DSS. Experiments were performed at 100 kHz MAS using 0.7 mm Bruker Biospin triple resonance probeheads. Sample temperatures varied between 19 and 27 °C and are specified for each experiment in *SI Appendix*, Table S7. They were determined using the relationship T(°C) = 455 – 90·δ(H_2_O) where δ(H_2_O) is the chemical shift of the supernatant water signal (55) internally referenced to DSS.

The MISSISSIPPI scheme (56) was used for solvent suppression in the CP-based 2D hNH and hCH as well as all CP-based 3D experiments, while no solvent suppression was used in the INEPT-based hNH and hCH experiments. A complete list of the recorded spectra and the corresponding experimental parameters are given in *SI Appendix*, Table S7 (CP-based 2D hNH, 2D hCH, 3D hCCH-TOBSY, 3D hNCAHA, and INEPT-based 2D hNH and 2DhCH, 2D hChH-MIRROR with reverse MIRROR H–H mixing scheme, and 2D HH-SD with spin diffusion H–H mixing scheme). CP-based 1D hnH and 1D hcH spectra of ^2^H^13^C^15^N-ORF7b and ORF7b^AFL^ were recorded with the exactly same parameters as the corresponding 2D spectra in 1D version, except the number of scans, which was set to 128 for all 1D spectra. 3D spectra were drift corrected by monitoring the water resonance frequency and using the SAFR scheme (57) and 2D hChH-MIRROR of ORF7b^F:L^ was drift corrected by linear drift correction (58). ^1^H detected bulk T_2_′ and T_1ρ_ measurements were recorded at 850 MHz using an hNH or hCH CP filter. CP duration was set according to the 2D hNH and 2D hCH parameter set. 19 delay times up to 10 ms were used for measuring T_2_′ and 16 for T_1ρ_. All experiments used a 13 kHz spin-lock.

2D hChH spectra using reverse MIRROR (59) as proton transfer sequence were recorded at a proton frequency of 1.2 GHz (except for the ERGIC lipid sample, that was recorded at 850 MHz) and with the pulse sequence shown in *SI Appendix*, Fig. S11. The rf field strength for the ^13^C spin lock in the MIRROR scheme was set to 6.6 kHz at 1.2 GHz and 4.7 kHz at 850 MHz corresponding to the chemical-shift difference of the ^1^H^γ^ of Leu and aromatic ^1^H-s of the Phe residues. To compensate for the lower amount of sample in the rotor, 1,280 scans were used for the ORF7b^L:F^ and E-cadTM^L^:ORF7b^F^ mixtures, while 320 scans were used for the control experiments.

The spectroscopic data were processed using Topspin 3.5 and 4.1 (Bruker Biospin) with zero filling to double the number of data points [except for 3D hNCAHA, where three to five times the data points were used) and a shifted sine-bell apodization function in the direct and indirect dimensions with SSB = 3 (2D hNH, 2D INEPT and 3D hNCAHA spectra) or 2 (2D hCH and 3D hCCH-TOBSY spectra)]. Assignments were performed using CcpNmr Analysis 2.4.2 (60, 61). For the relaxation analysis, peak intensities were exported to Microcal Origin (Origin Pro 8) and nonlinear fitting of the corresponding exponential decays was used. ^1^H homogeneous linewidths were calculated from the experimental relaxation times by the following formula: Δ^homo^ = 1/(π·T_2_′), while the ^1^H total linewidths were obtained by the CcpNmr Analysis software (61) using the parabolic approach (Δ^total^) for the Ala15 C–H crosspeak in 2D hCH spectra of each sample. ^1^H inhomogeneous linewidths were calculated as the difference of the total and the homogeneous linewidth: Δ^inhomo^ = Δ^total^ – Δ^homo^. The statistical errors were calculated from the SD obtained from Origin software and calculated by error propagation.

## Supplementary Material

Appendix 01 (PDF)

## Data Availability

All study data are included in the article and/or *SI Appendix*.
