# Supplementary material for: Analysis of the structure and interactions of the SARS-CoV-2 ORF7b accessory protein
Source: Proc Natl Acad Sci U S A. 2024 Nov 7;121(46):e2407731121. doi: 10.1073/pnas.2407731121 (PMC11573672; doi:10.1073/pnas.2407731121)
Supplement: Supplementary file 1 — Appendix 01 (PDF) [file pnas.2407731121.sapp.pdf]

PNAS

Supporting Information for
Analysis of the structure and interactions of the SARS-CoV-2 ORF7b accessory protein

Minh-Ha Nguyen[1,#], Gyula Palfy[2,#], Marie-Laure Fogeron[1], Marti Ninot Pedrosa[1], Johannes Zehnder[2], Vaclav Rimal[2], Morgane Callon[1,2], Lauriane Lecoq[1], Alexander Barnes[2], Beat H. Meier[2*], Anja Böckmann[1*]

Correspondence: Beat H. Meier, beme@ethz.ch; Anja Böckmann, a.bockmann@ibcp.fr

This PDF file includes:

Figures S1 to S15
Tables S1 to S8

A

```
TR|E0XIZ9|E0XIZ9_9BETC         MIHLTLFDFYLCVLSLLLFLVIIMLIIFCFVLELQDLNEQ------------ 40
TR|A0A166ZLE2|A0A166ZLE2_9NIDO MNELTLIDFYLCFLAFLLFLVLIMLIIFWFSLELQDIEEPCNKVFETYRSVL 52
TR|A0A0K1Z050|A0A0K1Z050_SARS  MNELTLIDFYLCFLAFLLFLVLIMLIIFWFSLELQDIEEPCNKV-------- 44
SP|Q3LZX6|NS7B_BCHK3           MNELTLIDFYLCFLAFLLFLVLIMLLIFWFSLEIQDIEEPCNKV-------- 43
TR|A0A6B9WFL9|A0A6B9WFL9_SARS2 MIELSLIDFYLCFLAFLLFLVLIMLIIFWFSLELQDHNETCHA--------- 44
                               *  *:*:*****.*::*****:***:** * **:** :*
```

B

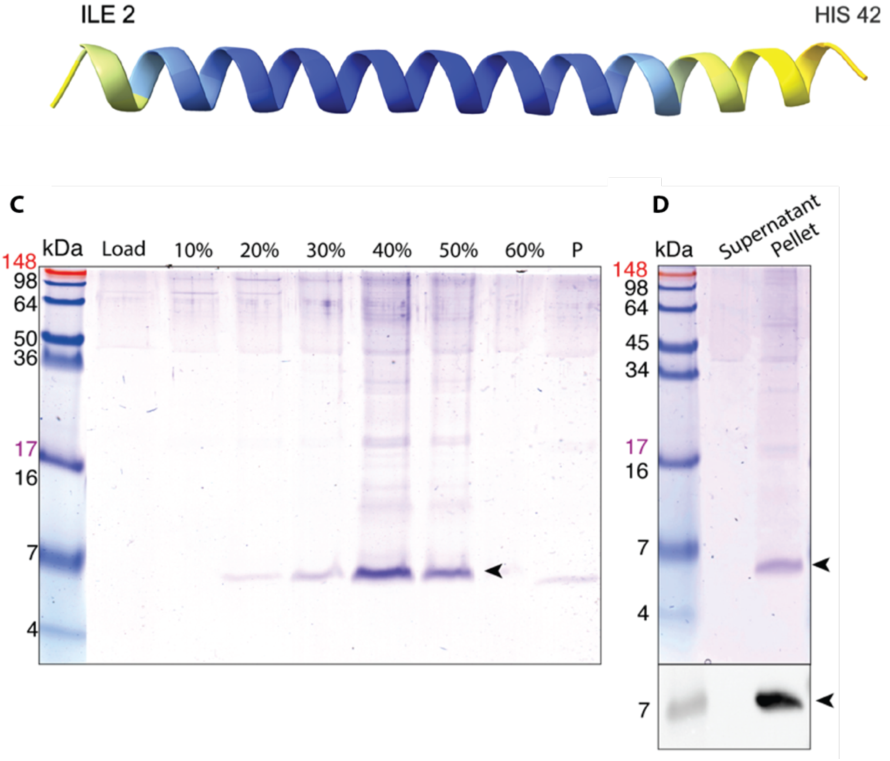

Figure S1: **The ORF7b protein**. (A) ORF7b sequence alignments of SARS-CoV and bat coronaviruses. E0XIZ9, bat coronavirus BM48-31/BGR/2008; A0A166ZLE2, bat coronavirus; A0A0K1Z050, bat SARS-like coronavirus YNLF_31C; A0A6B9WFL9, severe acute respiratory syndrome coronavirus 2 (SARS-CoV-2 F); NS7B_BCHK3, bat coronavirus HKU3 (BtCoV) (SARS-like coronavirus HKU3). (B) AlphaFold monomer model predicted for ORF7b (1-43), with alpha helix predicted from residue Ile2 to His42. Color coding is according to AlphaFold, as described in the caption to Figure 2. (C) Preparation of ORF7b samples for solid-state NMR using wheat germ cell-free protein synthesis (WG-CFPS). After synthesis, purification (Altincekic et al., 2021), and lipid reconstitution, ORF7b (black arrow head) was isolated on a 10-60% sucrose gradient (C), diluted to 8% of sucrose and sedimented (D) at 200000 g in home-made tools (Böckmann et al., 2009) into 0.7 mm NMR rotors.

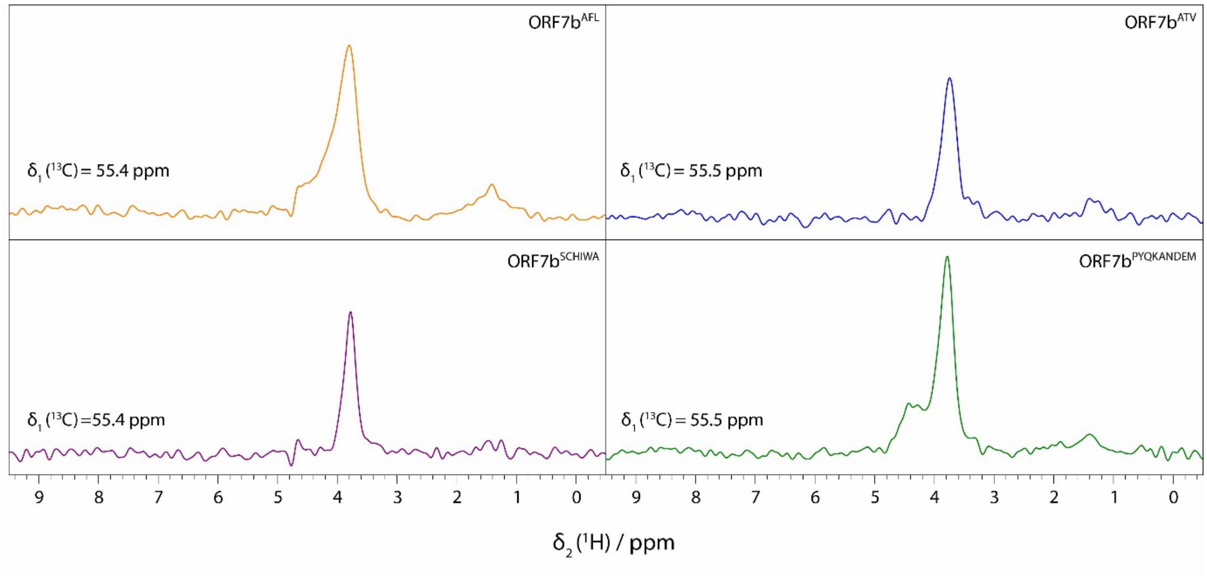

Figure S2: Ala 1D ^{1}H traces for Ala15 from 2D hCH spectra of ORF7b^{AFL} (orange), ORF7b^{ATV}, ORF7b^{SCHIWA}, and ORF7b^{PYQKANDEM}. The proton FWHH are 243 Hz, 250 Hz, 195 Hz, 254 Hz, respectively, but the lineshape is not well described by a Lorentzian/Gaussian.

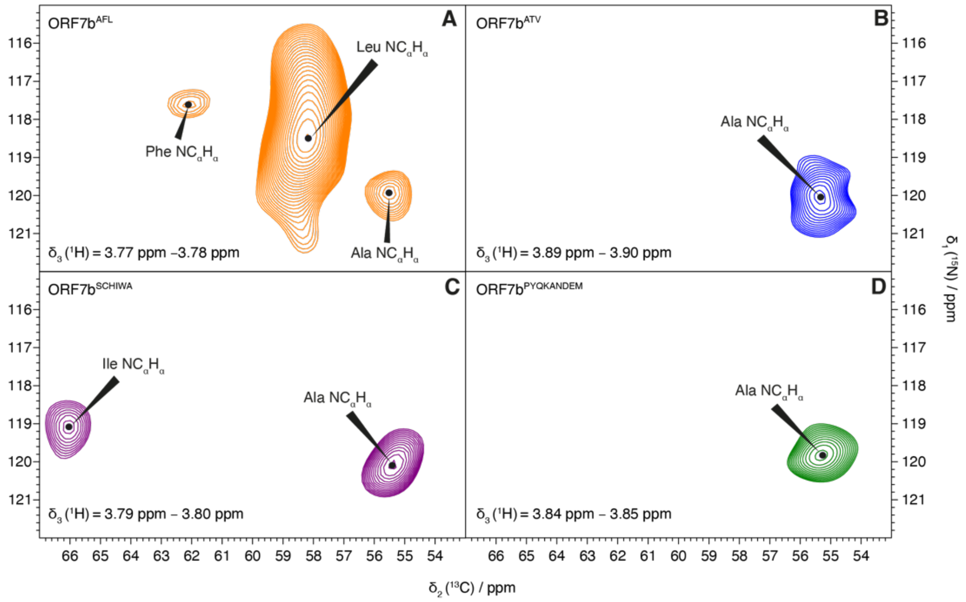

Figure S3: 2D CN planes of 3D hNCH spectra of selectively labelled ORF7b samples. ORF7b^{AFL} (orange), ORF7b^{ATV} (blue), ORF7b^{SCHIWA} (purple), ORF7b^{PYQKANDEM} (green). The Ala linewidth are 329 Hz, 378 Hz, 400 Hz, 434 Hz for $^{13}C^{\alpha}$, and 127 Hz, 118 Hz, 157 Hz, 119 Hz for ^{15}N for ORF7b^{AFL}, ORF7b^{ATV}, ORF7b^{SCHIWA}, and ORF7b^{PYQKANDEM}, respectively.

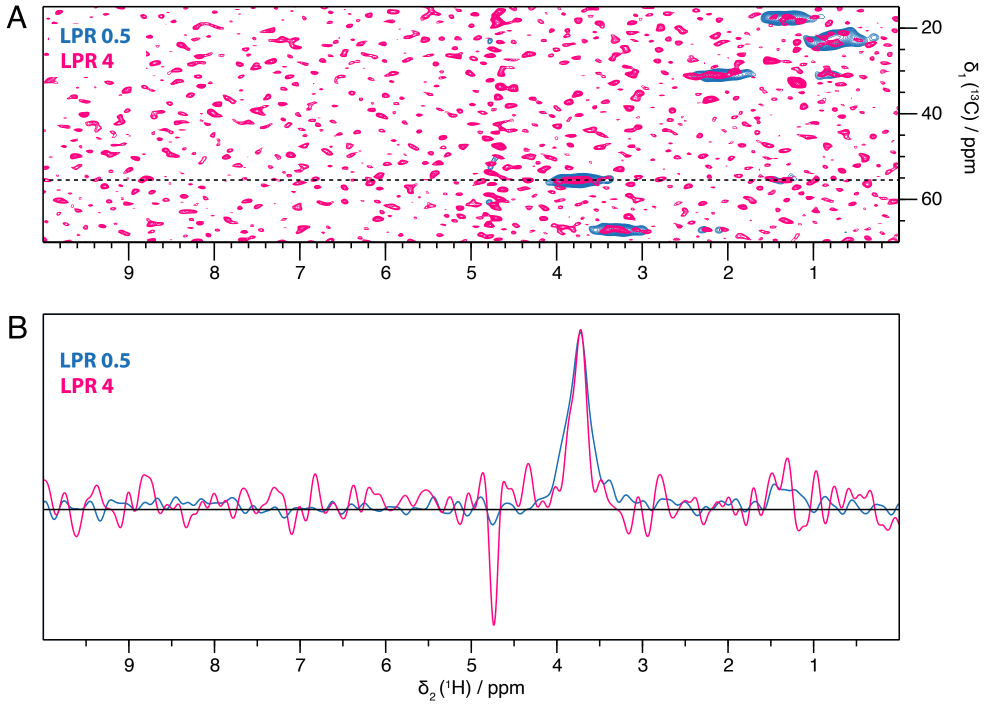

Figure S4: Comparison of spectra from ORF7b^{ATV} preparations with two different lipid-to-protein (LPR) ratios. (A) 2D hCH spectra of LPR 0.5 (blue) and LPR 4.0 (pink) and (B) 1D traces extracted at the alanine Cα resonance (dotted line in A). Spectra were recorded at 850 MHz with a sample temperature of 20-22 °C and under 100 kHz MAS in a 0.7 mm probe. It can be seen that the linewidth of the protein resonances are not significantly altered by the higher lipid-to-protein ratio: the line widths are 238 Hz (LPR 0.5) and 203 Hz (LPR 4), and thus differ by 35 Hz. The spectra are plotted at a signal level similar to the one shown for ORF7b^{ATV} LPR 0.5 below.

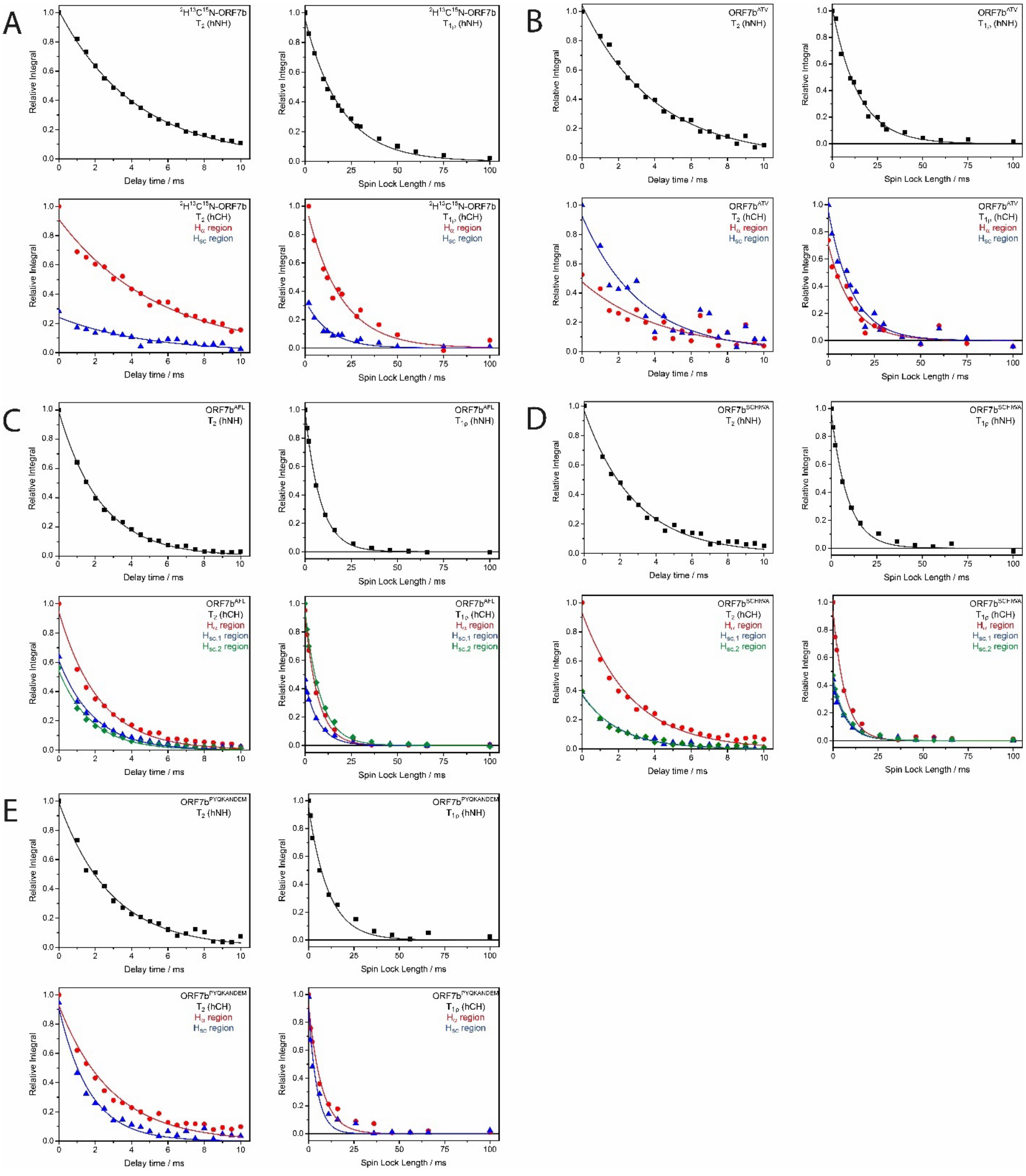

Figure S5: Relaxation analysis. Relaxation curves (hnH-edited 1D ^{1}H-T$_2$', hnH-edited 1D ^{1}H-T$_{1\rho}$, hcH-edited 1D ^{1}H-T$_2$' and hcH-edited 1D ^{1}H-T$_{1\rho}$) for the differently labelled ORF7b samples: (A) ^{2}H^{13}C^{15}N-ORF7b, (B) ORF7b^{ATV}, (C) ORF7b^{AFL}, (D) ORF7b^{SCHIWA}, (E) ORF7b^{PYQKANDEM} recorded at 850 MHz at 20-27 °C (the exact temperatures as well as the parameters for CP transfer are shown in Table S8 for each sample). The curves used for hnH-edited spectra are black (6 – 10 ppm region), for hcH-edited spectra they are red (H$^\alpha$ region: 3.1 – 4.8 ppm), blue and green (H^{sc} region:

-0.6 – 2.7 ppm, in some cases the entire region was used shown in blue, in other cases when the region could be split into two minima, two different regions were fitted separately shown in blue and green).

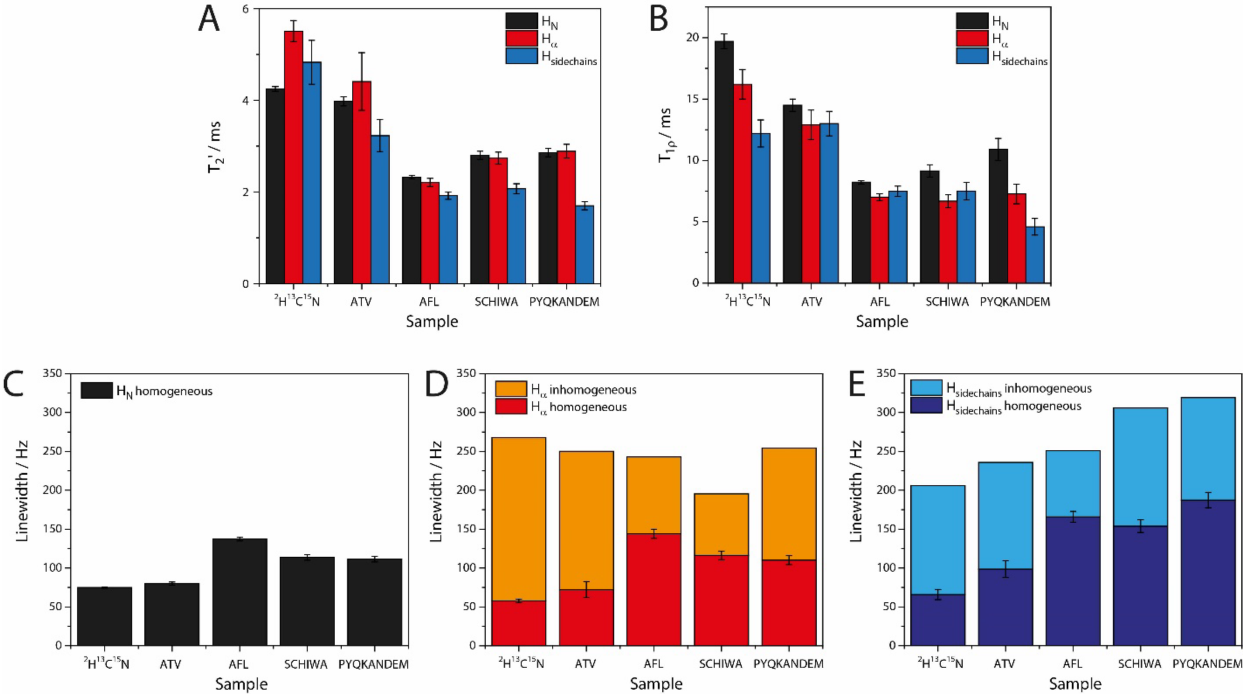

Figure S6. Relaxation times of ORF7b. (A) Bulk ^{1}H-T$_2$' and (B) ^{1}H-T$_{1\rho}$ relaxation times for the differently labeled samples (^{2}H^{13}C^{15}N-ORF7b, ORF7b^{ATV}, ORF7b^{AFL}, ORF7b^{SCHIWA}, and ORF7b^{PYQKANDEM}) and corresponding linewidths. The bulk relaxation times were separately evaluated for three different spectral regions, namely the amide protons (6 – 10 ppm), the Hα 3.1 – 4.8 ppm) and the sidechain protons (-0.6 – 2.7 ppm). The differences in ^{1}H-T$_2$' and ^{1}H-T$_{1\rho}$ between the samples mainly arise from the different proton density, since unobserved residues are deuterated on aliphatic and aromatic protons. The homogeneous linewidths (C) were calculated from T$_2$' values in the corresponding regions, the total linewidths were measured on the spectra by CCPN v2. software based on the peak of the Ala15 in the 2D hCH spectra, and the inhomogeneous linewidths (D,E) were estimated by the difference of the total and the homogeneous linewidths.

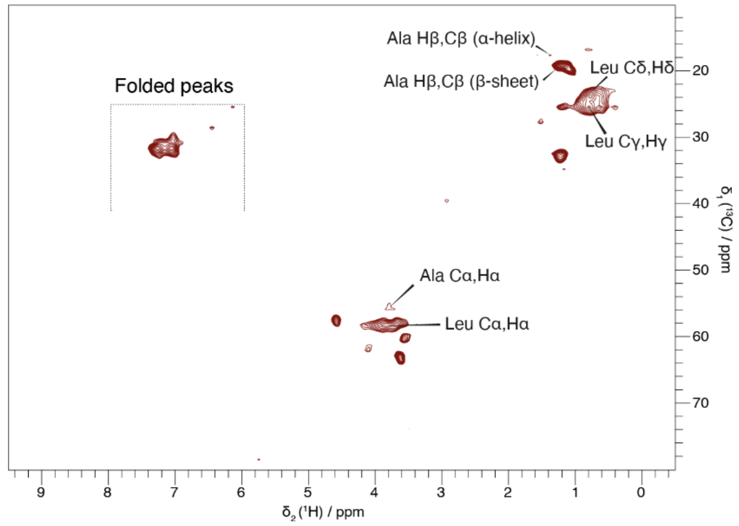

Figure S7: INEPT-hCH spectrum. Tentative assignment of ORF7b^{AFL} are shown.

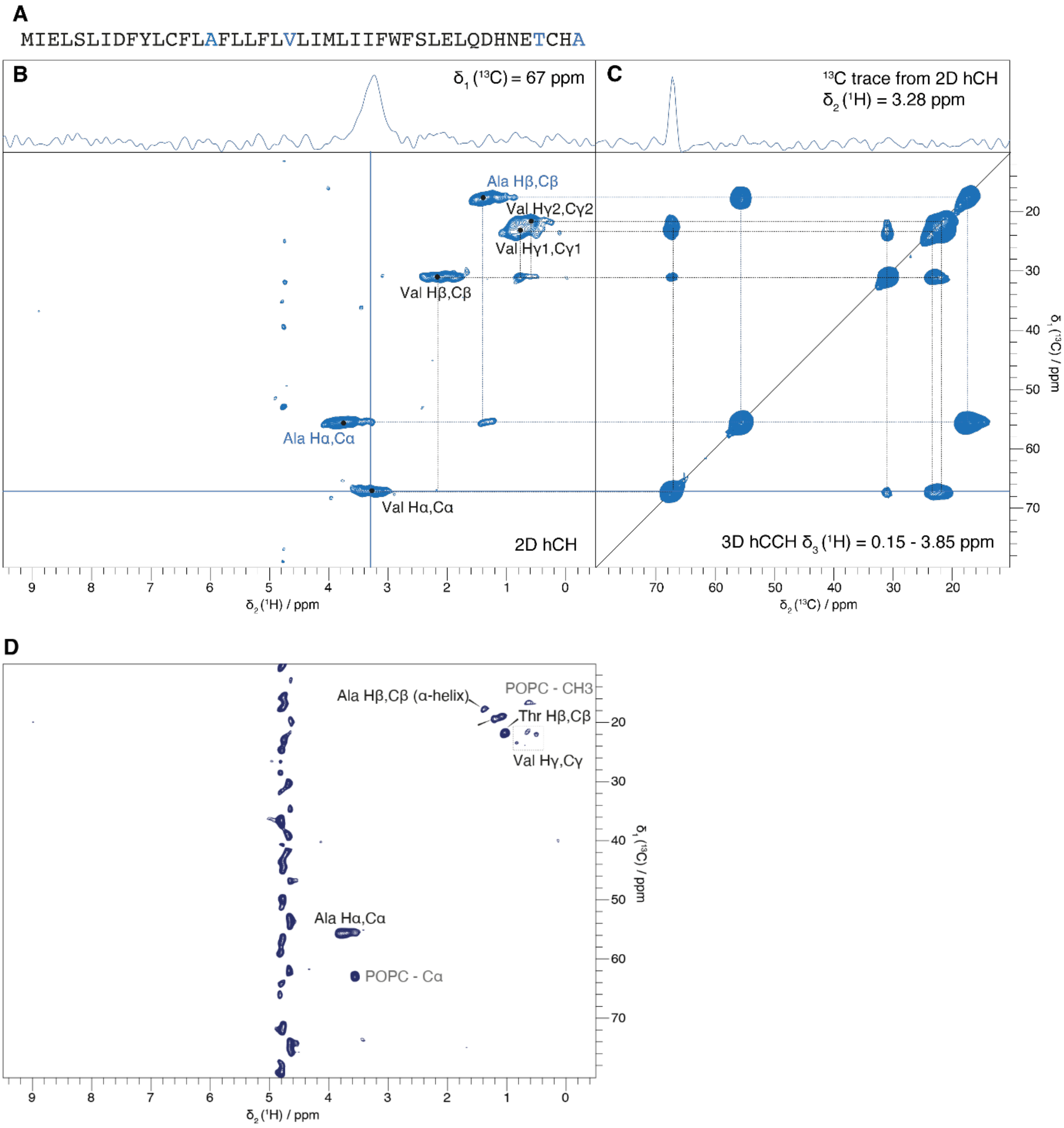

Figure S8: Spectra recorded on ORF7b^{ATV}. (A) Amino-acid labeling of ORF7b. (B) 2D hCH spectrum of ORF7b^{ATV}, with a 1D extract shown at 67 ppm. Assignments of the signals to the different amino acids are given, in black for Val, and in blue for Ala. (C) 3D hCCH spectrum, all planes between 0.15 and 3.85 ppm are shown. (D) INEPT-hCH spectrum with tentative assignment.

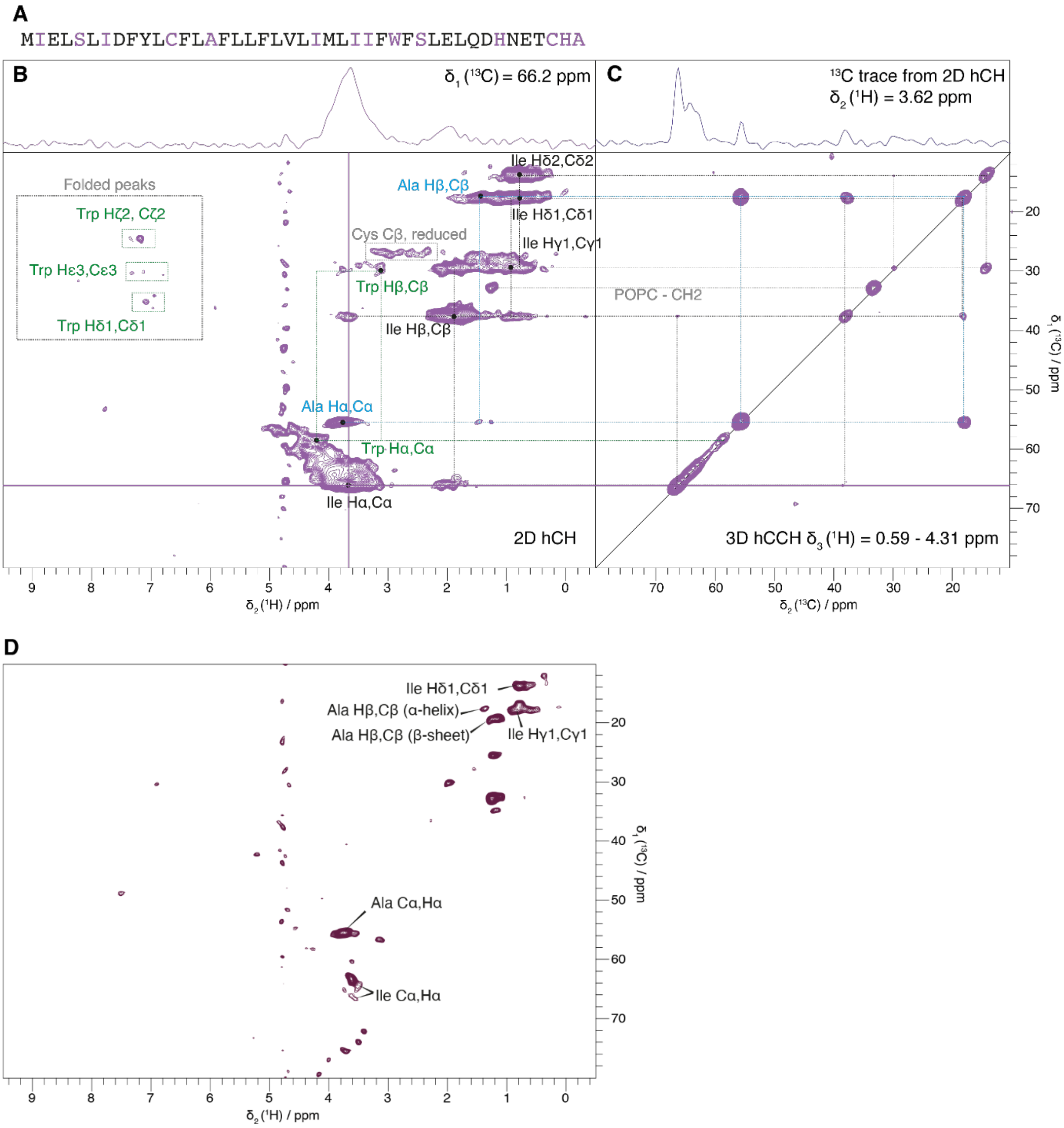

Figure S9: Spectra recorded on ORF7b^{SCHIWA}. (A) Amino-acid labelling of ORF7b. B) 2D hCH spectrum of ORF7b^{SCHIWA}, with a 1D extract shown at 67 ppm. Assignments of the signals to the different amino acids are given, in black for Ile, in blue for Ala, in green for Trp, and in grey for Cys. C) 3D hCCH spectrum, all planes between 0.59 and 4.31 ppm are shown. (D) INEPT-hCH spectrum with tentative assignment.

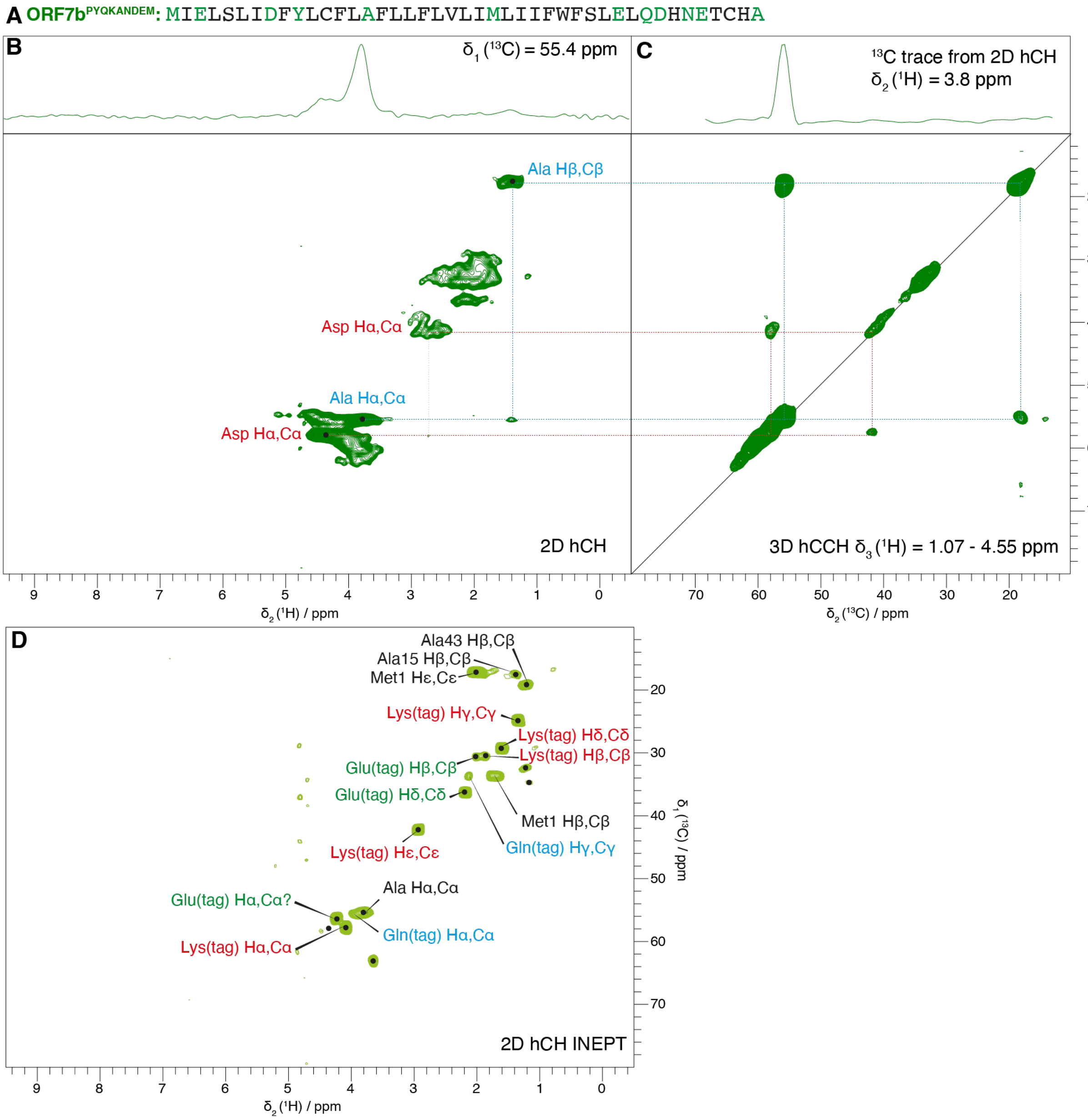

Figure S10: Spectra recorded on ORF7b^{PYQKANDEM}. (A) Amino-acid labelling of ORF7b. (B) 2D hCH spectrum of ORF7b^{PYQKANDEM}, with a 1D extract shown at 55.4 ppm. Assignments of the signals to the different amino acids are given. (C) 3D hCCH spectrum, all planes between 1.07 and 4.55 ppm are shown. (D) INEPT-hCH spectrum with tentative assignment.

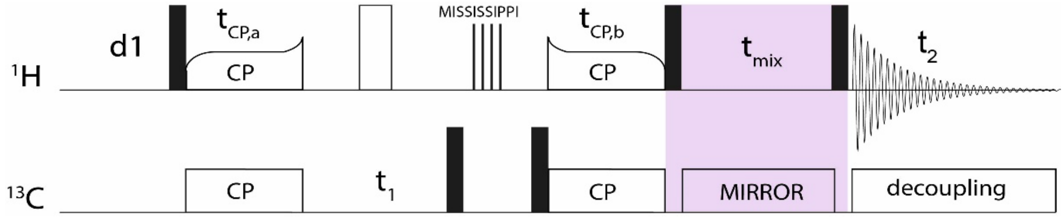

Figure S11: 2D hChH-MIRROR. The schematic pulse sequence is shown. For parameters used see Table S7.

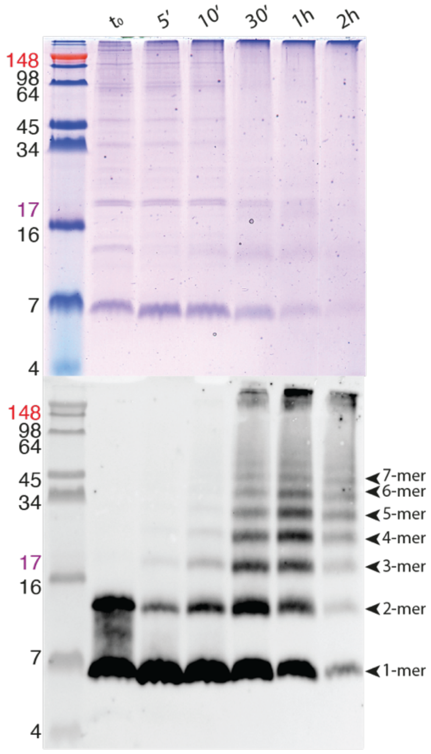

Figure S12: Cross linking of ORF7b in DDM detergent. Cross linking experiments show that with increasing incubation time in presence of 4-(4,6-dimethoxy-1,3,5-triazin-2-yl)-4-methylmorpholinium chloride (DMTMM), a zero-length crosslinking agent, the amount of ORF7b monomer (1-mer) in DDM decreases. After 1 hour of incubation, the tetrameric (4-mer) and pentameric (5-mer) forms are the most abundant multimeric species, as well as higher aggregates found in the gel stacks.

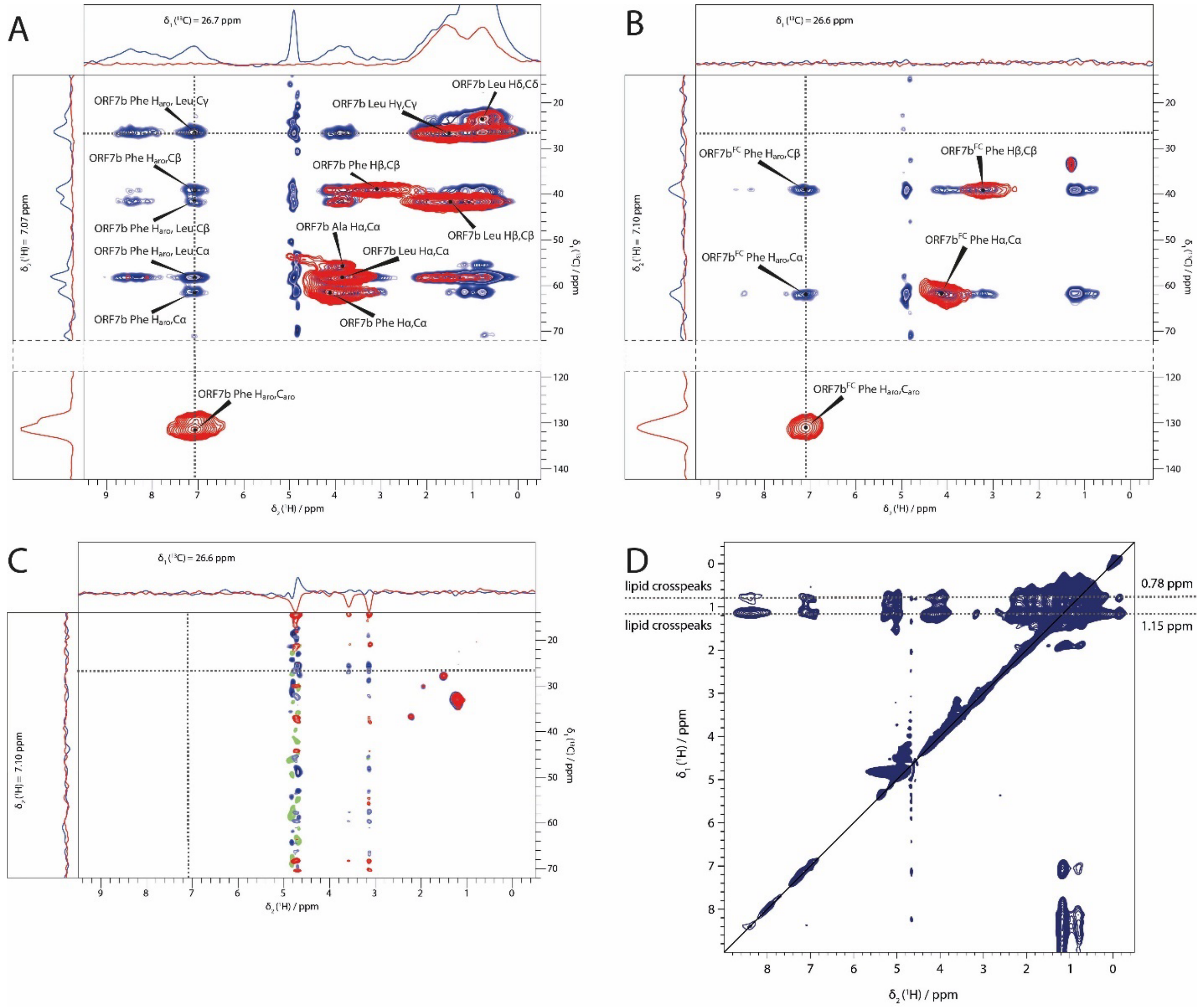

Figure S13. Control experiments for ORF7b homologous interactions. (A) First, we excluded that the cross peak could arise from a correlation with lipids protons, by recording a 2D hChH spectrum with the same parameters for ORF7b^{AFL}, but this time using deuterated d82-POPC lipids. In the hChH spectrum in blue, superimposed for reference on a hCH spectrum in red, an intramolecular Phe-Leu contact can still be detected, meaning that the cross peak do not originate from poorly filtered lipid protons localized near the Leu frequency. (B) Second, we measured ORF7b^{FC} to confirm that in absence of labelled Leu, no cross signals in the methyl region are observed in the hChH spectrum in blue, superimposed for reference on the hCH spectrum in red. (C) Finally, we measured a rotor filled only with ERGIC lipids, without protein, resulting in the hCH and hChH spectra shown in red and blue respectively, to make sure that no lipid-lipid cross peaks are observed. (D) 2D HH-SD spectra of E-cadTM^{L}:ORF7b^{F}. It can be seen that 2D HH spectra cannot be used to assess interactions, since large cross signals with lipids are indeed observed if no ^{13}C filter is applied as in a hChH. The Leu Cγ frequency is marked by a dotted line in all panels, to show that no signal is observed at this ^{13}C frequency in ORF7b^{FC} or ERGIC lipids.

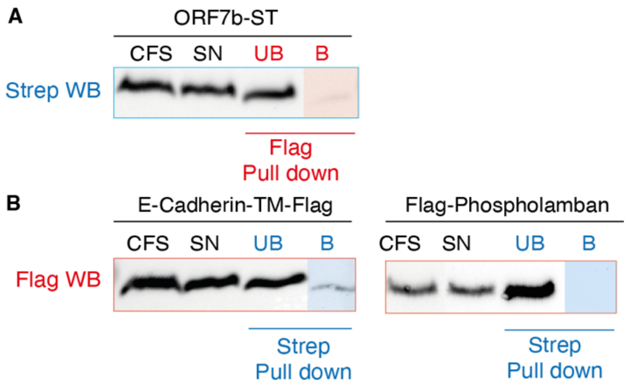

Figure S14: Control experiments for the ORF7b + PLN / E-cadTM pull-down assay. Each interactant was produced alone and was cross pulled down, (A) Flag pull down for Strep-tagged ORFb and Strep pull down for Flag tagged E-cadTM or PLN (B). No unspecific binding of the interactants was observed, as everything was found in the unbound fractions.

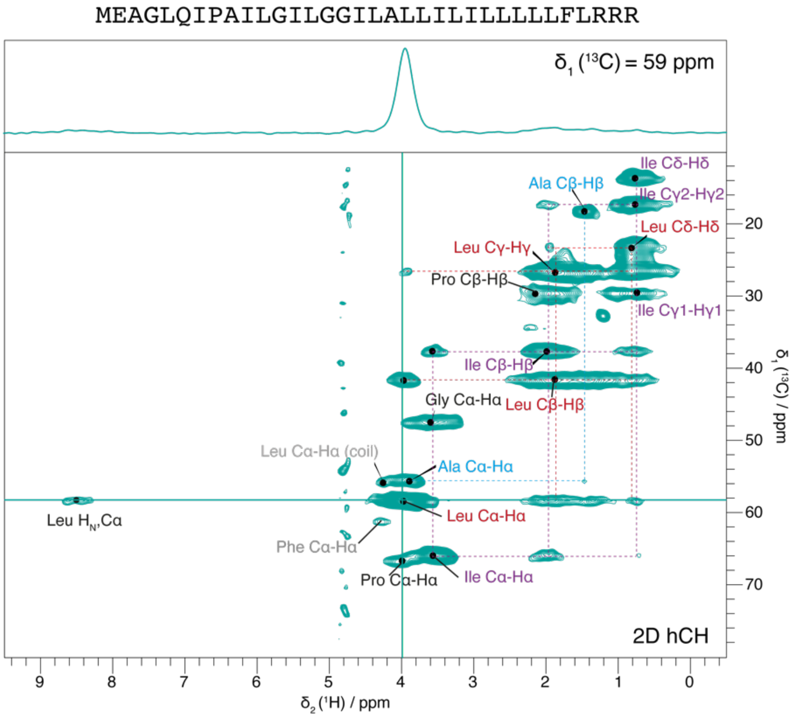

Figure S15: E-cadTM 2D hCH. Assignments to amino-acid types are given.

Table S1: Intramolecular distances in the PLN structure (2KYL, first structure), as well as used upper and lower distance bounds used for the CYANA calculation of the ORF7b model. The upper and lower distance bounds were set to the distance from the 2KYL ± 0.25 Å.

Contact in 2KYV Experimental structure	Distance/ Å	Corresponding contact in Orf7b model	upper distance bound / Å	lower distance bound / Å
A 33I Cα – A 40I Cα	10.56	A 7I Cα – A 14L Cα	10.81	10.31
A 33I Cβ – A 40I Cβ	10.65	A 7I Cβ – A 14L Cβ	10.90	10.40
A 40I Cα – A 47I Cα	10.19	A 14L Cα – A 21V Cα	10.44	9.94
A 40I Cβ – A 47I Cβ	10.14	A 14L Cβ – A 21V Cβ	10.39	9.89
A 30N Cα – A 37L Cα	10.38	A 4L Cα – A 11L Cα	10.63	10.13
A 30N Cβ – A 37L Cβ	10.35	A 4L Cβ – A 11L Cβ	10.60	10.10
A 37L Cα – A 44L Cα	10.45	A 11L Cα – A 18L Cα	10.70	10.20
A 37L Cβ – A 44L Cβ	10.44	A 11L Cβ – A 18L Cβ	10.69	10.19
A 44L Cα – A 51L Cα	10.30	A 18L Cα – A 25L Cα	10.55	10.05
A 44L Cβ – A 51L Cβ	10.57	A 18L Cβ – A 25L Cβ	10.82	10.32

Table S2: Measured intermolecular distances in the PLN structure (2KYL, first structure) and used upper and lower distance limits for the CYANA calculation of the ORF7b model.

Contact in 2KYV	Distance / Å	Corresponding contact in ORF7b	upl / Å	lol / Å
A 29Q Cα – B 29Q Cα	8.59	A 3E Cα – B 3E Cα	8.84	8.34
A 29Q Cα – C 29Q Cα	13.91	A 3E Cα – C 3E Cα	14.16	13.66
A 30N Cα – B 30N Cα	7.88	A 4L Cα – B 4L Cα	8.13	7.63
A 30N Cβ – B 30N Cβ	8.38	A 4L Cβ – B 4L Cβ	8.63	8.13
A 31L Cα – B 31L Cα	11.72	A 5S Cα – B 5S Cα	11.97	11.47
A 32F Cα – B 32F Cα	11.17	A 6L Cα – B 6L Cα	11.42	10.92
A 33I Cα – B 33I Cα	7.02	A 7L Cα – B 7L Cα	7.27	6.77
A 33I Cβ – B 33I Cβ	5.60	A 7L Cβ – B 7L Cβ	5.85	5.35
A 34N Cα – B 34N Cα	9.17	A 8D Cα – B 8D Cα	9.42	8.92
A 35F Cα – B 35F Cα	12.02	A 9F Cα – B 9F Cα	12.27	11.77
A 36C Cα – B 36C Cα	9.36	A 10Y Cα – B 10Y Cα	9.61	9.11
A 37L Cα – B 37L Cα	6.81	A 11L Cα – B 11L Cα	7.06	6.56
A 37L Cβ – B 37L Cβ	6.38	A 11L Cβ – B 11L Cβ	6.63	6.13
A 38I Cα – B 38I Cα	10.90	A 12C Cα – B 12C Cα	11.15	10.65
A 39L Cα – B 39L Cα	11.32	A 13F Cα – B 13F Cα	11.57	11.07
A 40I Cα – B 40I Cα	7.23	A 14L Cα – B 14L Cα	7.48	6.98
A 40I Cβ – B 40I Cβ	5.66	A 14L Cβ – B 14L Cβ	5.91	5.41
A 41C Cα – B 41C Cα	9.10	A 15A Cα – B 15A Cα	9.35	8.85
A 42L Cα – B 42L Cα	12.25	A 16F Cα – B 16F Cα	12.50	12.00
A 43L Cα – B 43L Cα	9.73	A 17L Cα – B 17L Cα	9.98	9.48
A 44L Cα – B 44L Cα	7.35	A 18L Cα – B 18L Cα	7.60	7.10
A 44L Cβ – B 44L Cβ	6.86	A 18L Cβ – B 18L Cβ	7.11	6.61
A 45I Cα – B 45I Cα	11.56	A 19F Cα – B 19F Cα	11.81	11.31
A 46C Cα – B 46C Cα	12.12	A 20L Cα – B 20L Cα	12.37	11.87
A 47I Cα – B 47I Cα	8.09	A 21V Cα – B 21V Cα	8.34	7.84
A 47I Cβ – B 47I Cβ	6.54	A 21V Cβ – B 21V Cβ	6.79	6.29
A 48I Cα – B 48I Cα	9.55	A 22L Cα – B 22L Cα	9.80	9.30
A 49V Cα – B 49V Cα	13.04	A 23I Cα – B 23I Cα	13.29	12.79
A 50M Cα – B 50M Cα	11.45	A 24M Cα – B 24M Cα	11.70	11.20
A 51L Cα – B 51L Cα	7.98	A 25L Cα – B 25L Cα	8.23	7.73
A 51L Cβ – B 51L Cβ	6.58	A 25L Cβ – B 25L Cβ	6.83	6.33

A 52L Cα – B 52L Cα	11.31	A 26I Cα – B 26I Cα	11.56	11.06
A 52L Cα – C 52L Cα	18.32	A 26I Cα – C 26I Cα	18.57	18.07

Table S3: ERGIC-mimetic membrane lipid composition

	MW (g/mol)	Molar ratio (%)	Mass ratio (%)
**POPC**	760	45	41.7
**POPE**	718	20	20.8
**PI**	902	13	16.7
**POPS**	784	7	10.4
**Chol**	387	15	10.4

Table S4: Relaxation data of the $^{2}H^{13}C^{15}N$ and the selectively labeled samples. All measurements were done at 850 MHz and bulk homogeneous, total and inhomogenous linewidths.

	T_2' (hNH) / ms	T_2' (hCH) / ms for H_α	T_2' (hCH) / ms for H_{sc}	$T_{1\rho}$ (hNH) / ms	$T_{1\rho}$ (hCH) / ms for H^α	$T_{1\rho}$ (hCH) / ms for H^{sc}
**$^{2}H^{13}C^{15}N$-ORF7b**	4.25 ± 0.05	5.5 ± 0.3	4.8 ± 0.5	19.7 ± 0.6	16.2 ± 1.2	12.2 ± 1.1
**$ORF7b^{ATV}$**	3.98 ± 0.10	4.4 ± 0.7	3.2 ± 0.4	14.5 ± 0.5	12.9 ± 1.2	13.0 ± 1.0
**$ORF7b^{AFL}$**	2.32 ± 0.04	2.21 ± 0.09	1.92 ± 0.08	8.23 ± 0.14	7.0 ± 0.3	7.5 ± 0.5
**$ORF7b^{SCHIWA}$**	2.80 ± 0.09	2.74 ± 0.13	2.07 ± 0.11	9.2 ± 0.5	6.7 ± 0.6	7.5 ± 0.8
**$ORF7b^{PYQKANDEM}$**	2.86 ± 0.09	2.89 ± 0.15	1.70 ± 0.09	10.9 ± 0.9	7.3 ± 0.8	4.6 ± 0.7

	**Linewidth homogeneous / Hz**			**Linewidth inhomogeneous / Hz**			**Linewidth total / Hz**		
	**NH**	**H^α**	**H^{sc}**	**NH**	**H^α**	**H^{sc}**	**NH**	**H^α**	**H^{sc}**
**$^{2}H^{13}C^{15}N$-ORF7b**	74.9 ± 0.9	58 ± 3	66 ± 7	-	210	140	-	268	206
**$ORF7b^{ATV}$**	80 ± 2	72 ± 11	99 ± 11	-	178	137	-	250	236
**$ORF7b^{AFL}$**	137 ± 3	144 ± 6	166 ± 7	-	99	85	-	243	251
**$ORF7b^{SCHIWA}$**	114 ± 4	116 ± 6	154 ± 9	-	79	152	-	195	306
**$ORF7b^{PYQKANDEM}$**	111 ± 4	110± 6	187 ± 10	-	144	132	-	254	319

Table S5: Chemical shift of observed ORF7b residues/spin systems

	Chemical Shift /ppm															
	Ca	Cb	Cd	Cd1/2	Cg	Cg1	Cg2	Cz	Cz2	Ha	Hb	Hd1/2	Hg	Hg1	Hg2	Hz
**D8**	57.5	41.4								2.73	4.42					
**C12**		27.2									2.99					
**A15**	55.6	17.6								3.76	1.39					
**V21**	67.2	31.0			21.8					3.24	2.09		0.74			
**W29**	58.4	29.3							114.4	4.21		2.04				7.19
**F30**	57.4									4.58						
**F9,13,16,19,28**	61.4	39.0	131.3					128.9		4.08	3.07	7.10				6.96
**L11,14,17,18,20,22,25**	58.2	41.6		23.4	26.9					3.79	3.85	0.71	1.52			
**I23,26,27**	66.3	37.6	13.8			29.6	17.6			3.65	2.04	0.76		1.95	0.78	
**E52**	56.3	30.5	36.2							4.22	2.08	2.20				
**K53**	57.9	30.4	29.2		24.9					4.09	1.898	1.60	1.38			

Table S6: ORF7b secondary chemical shifts

	Secondary Chemical shifts	
	**ΔδCα**	**ΔδCβ**
**D8**	3.41	0.64
**C12**		-2.59
**A15**	0.28	-1.43
**V21**	5.40	-1.73
**W29**	0.82	0.04
**F30**	0.48	
**F9,13,16,19,28**	4.46	-0.43
**L11,14,17,18,20,22,25**	3.35	-0.27
**I23,26,27**	5.68	-0.63
**E52**	-0.09	0.48
**K53**	1.5	-2.17

Table S7: List of experiments and parameters for ORF7b NMR samples

$^{2}H^{13}C^{15}N$-ORF7b

Experiment	2D hNH CP	2D hCH
^{1}H Field / MHz	850	850
MAS frequency / kHz	100	100
Sample Temperature / °C	21	21
Rotor diameter / mm	0.7	0.7
Number of scans	128	248
Experimental time / h	8.3h	16.1
t1 increment	170	170
Sweep width (t1) / ppm	80	100
Acquisition time (t1) / ms	12.3	4.0
t2 increment	3072	3072
Sweep width (t2) / ppm	46.7	46.7
Acquisition time (t2) / ms	38.7	38.7
^{1}H decoupling (swftppm) / kHz	10	10
^{13}C decoupling (WALTZ64) / kHz	-	5
^{15}N decoupling (WALTZ64) / kHz	5	-
MISSISSIPPI (120 ms) / kHz	20	20
Interscan delay / s	1.2	1.2
**Transfer 1**	**HN (dipolar)**	**HC (dipolar)**
^{1}H field / kHz	80.0	80.0
^{13}C field / kHz	-	16.6
^{15}N field / kHz	13.5	-
Shape	Tangent ^{1}H	Tangent ^{1}H
CP contact time / ms	1.6	1.0
**Transfer 2**	**NH (dipolar)**	**CH (dipolar)**
^{1}H field / kHz	80.0	80.0
^{13}C field / kHz	-	16.6
^{15}N field / kHz	13.5	-
Shape	Tangent ^{1}H	Tangent ^{1}H
CP contact time / ms	1.2	1.0
Carrier ^{1}H / ppm	4.7	4.7
Carrier ^{13}C / ppm	-	55.0
Carrier ^{15}N / ppm	117.5	-
**Figure n°**	3A	-

ORF7b^{AFL}

Experiment	2D CP-hNH CP	2D CP-hCH	2D INEPT-hNH	2D INEPT-hCH
^{1}H Field / MHz	850	850	850	850
MAS frequency / kHz	100	100	100	100
Sample Temperature / °C	27	27	27	27
Rotor diameter / mm	0.7	0.7	0.7	0.7
Number of scans	64	64	64	64
Experimental time	4.1 h	4.1 h	4.2 h	4.2 h
t1 increment	170	170	170	170
Sweep width (t1) / ppm	80	100	40	100
Acquisition time (t1) / ms	12.3	4.0	24.7	4.0
t2 increment	3072	3072	3072	2048
Sweep width (t2) / ppm	46.7	46.7	46.7	46.7
Acquisition time (t2) / ms	38.7	38.7	38.7	25.8
^{1}H decoupling (swftppm) / kHz	10	10	-	-
^{13}C decoupling (WALTZ64) / kHz	-	5	-	5
^{15}N decoupling (WALTZ64) / kHz	5	-	5	5
MISSISSIPPI (120 ms) / kHz	20	20	20	20
Interscan delay / s	1.2	1.2	1.2	1.2
**Transfer 1**	**HN (dipolar)**	**HC (dipolar)**	**HN (INEPT)**	**HC (INEPT)**
^{1}H field / kHz	80.0	80.0	-	-
^{13}C field / kHz	-	19.2	-	-
^{15}N field / kHz	15.1	-	-	-
Shape	Tangent ^{1}H	Tangent ^{1}H	-	-
CP contact time / ms	1.0	0.8	-	-
INEPT delay (~1/4J) / ms	-	-	2.2	1.8
INEPT refocusing delay / ms	-	-	2.2	1.4
**Transfer 2**	**NH (dipolar)**	**CH (dipolar)**	**NH (INEPT)**	**CH (INEPT)**
^{1}H field / kHz	80.0	71.1	-	-
^{13}C field / kHz	-	19.2	-	-
^{15}N field / kHz	15.1	-	-	-
Shape	Tangent ^{1}H	Tangent ^{1}H	-	-
CP contact time / ms	1.1	0.7	-	-
INEPT delay / ms	-	-	2.2	1.8
INEPT refocusing delay / ms	-	-	2.2	1.4
Carrier ^{1}H / ppm	4.8	4.8	4.8	4.8
Carrier ^{13}C / ppm	-	40	-	40
Carrier ^{15}N / ppm	117.5	-	117.5	117.5
**Figure n°**	-	4D	-	S6

ORF7b^{ATV}

Experiment	2D CP-hNH CP	2D CP-hCH	2D INEPT-hNH	2D INEPT-hCH
^{1}H Field / MHz	850	850	850	850
MAS frequency / kHz	100	100	100	100
Sample Temperature / °C	24 (estimated)	24 (estimated)	24 (estimated)	24 (estimated)
Rotor diameter / mm	0.7	0.7	0.7	0.7
Number of scans	128	64	64	64
Experimental time	8.3 h	4.1 h	4.2 h	4.2 h
t1 increment	170	170	170	170
Sweep width (t1) / ppm	80	100	80	100
Acquisition time (t1) / ms	12.3	4.0	12.3	4.0
t2 increment	3072	3072	3072	4096
Sweep width (t2) / ppm	46.7	46.7	46.7	46.7
Acquisition time (t2) / ms	38.7	38.7	38.7	51.6
^{1}H decoupling (swftppm) / kHz	10	10	-	-
^{13}C decoupling (WALTZ64) / kHz	-	5	-	5
^{15}N decoupling (WALTZ64) / kHz	5	-	5	5
MISSISSIPPI (120 ms) / kHz	20	20	20	20
Interscan delay / s	1.2	1.2	1.2	1.2
**Transfer 1**	**HN (dipolar)**	**HC (dipolar)**	**HN (INEPT)**	**HC (INEPT)**
^{1}H field / kHz	80.0	80.0	**-**	**-**
^{13}C field / kHz	-	13.7	-	-
^{15}N field / kHz	12.8	-	-	-
Shape	Tangent ^{1}H	Tangent ^{1}H	-	-
CP contact time / ms	1.0	0.7	-	-
INEPT delay (~1/4J) / ms	-	-	2.2	1.8
INEPT refocusing delay / ms	-	-	2.2	1.4
**Transfer 2**	**NH (dipolar)**	**CH (dipolar)**	**NH (INEPT)**	**CH (INEPT)**
^{1}H field / kHz	80.0	80.0	**-**	**-**
^{13}C field / kHz	-	13.7	-	-
^{15}N field / kHz	12.8	-	-	-
Shape	Tangent ^{1}H	Tangent ^{1}H	-	-
CP contact time / ms	1.1	0.7	-	-
INEPT delay / ms	-	-	2.2	1.8
INEPT refocusing delay / ms			2.2	1.4
Carrier ^{1}H / ppm	4.8	4.8	4.8	4.7
Carrier ^{13}C / ppm	-	40	-	55
Carrier ^{15}N / ppm	117.5	-	117.5	117.5
**Figure n°**	-	S8B	**-**	S8D

ORF7b^{SCHIWA}

Experiment	2D CP-hNH CP	2D CP-hCH	2D INEPT-hNH	2D INEPT-hCH
^{1}H Field / MHz	850	850	850	850
MAS frequency / kHz	100	100	100	100
Sample Temperature / °C	27	27	27	27
Rotor diameter / mm	0.7	0.7	0.7	0.7
Number of scans	64	64	64	64
Experimental time	4.1 h	4.1 h	4.2 h	4.2 h
t1 increment	170	170	170	170
Sweep width (t1) / ppm	80	90	40	90
Acquisition time (t1) / ms	12.3	4.4	24.7	4.4
t2 increment	3072	3072	3072	3072
Sweep width (t2) / ppm	46.7	46.7	46.7	46.7
Acquisition time (t2) / ms	38.7	38.7	38.7	38.7
^{1}H decoupling (swftppm) / kHz	10	10	-	-
^{13}C decoupling (WALTZ64) / kHz	-	5	-	5
^{15}N decoupling (WALTZ64) / kHz	5	-	5	5
MISSISSIPPI (120 ms) / kHz	20	20	20	20
Interscan delay / s	1.2	1.2	1.2	1.2
**Transfer 1**	**HN (dipolar)**	**HC (dipolar)**	**HN (INEPT)**	**HC (INEPT)**
^{1}H field / kHz	80.0	80.0	-	-
^{13}C field / kHz	-	17.9	-	-
^{15}N field / kHz	13.3	-	-	-
Shape	Tangent ^{1}H	Tangent ^{1}H	-	-
CP contact time / ms	1.0	0.6	-	-
INEPT delay (1/4J) / ms	-	-	2.2	1.8
INEPT refocusing delay / ms	-	-	2.2	1.4
**Transfer 2**	**NH (dipolar)**	**CH (dipolar)**	**NH (INEPT)**	**CH (INEPT)**
^{1}H field / kHz	80.0	75.4	-	-
^{13}C field / kHz	-	17.9	-	-
^{15}N field / kHz	13.3	-	-	-
Shape	Tangent ^{1}H	Tangent ^{1}H	-	-
CP contact time / ms	1.1	0.5	-	-
INEPT delay (1/4J) / ms	-	-	2.2	1.8
INEPT refocusing delay / ms	-	-	2.2	1.4
Carrier ^{1}H / ppm	4.8	4.8	4.8	4.8
Carrier ^{13}C / ppm	-	45	-	45
Carrier ^{15}N / ppm	117.5	-	117.5	117.5
**Figure n°**	-	S9B	-	S9D

ORF7b^{PYQKANDEM}

Experiment	2D CP-hNH CP	2D CP-hCH	2D INEPT-hNH	2D INEPT-hCH
^{1}H Field / MHz	850	850	850	850
MAS frequency / kHz	100	100	100	100
Sample Temperature / °C	27	27	27	27
Rotor diameter / mm	0.7	0.7	0.7	0.7
Number of scans	224	248	64	64
Experimental time	14.7 h	16.1 h	4.2 h	4.2 h
t1 increment	170	170	170	170
Sweep width (t1) / ppm	80	100	80	100
Acquisition time (t1) / ms	12.3	4.0	12.3	4.0
t2 increment	3072	3072	3072	3072
Sweep width (t2) / ppm	46.7	46.7	46.7	46.7
Acquisition time (t2) / ms	38.7	38.7	38.7	38.7
^{1}H decoupling (swftppm) / kHz	10	10	-	-
^{13}C decoupling (WALTZ64) / kHz	-	5	-	5
^{15}N decoupling (WALTZ64) / kHz	5	-	5	5
MISSISSIPPI (120 ms) / kHz	20	20	20	20
Interscan delay / s	1.2	1.2	1.2	1.2
**Transfer 1**	**HN (dipolar)**	**HC (dipolar)**	**HN (INEPT)**	**HC (INEPT)**
^{1}H field / kHz	80.0	80.0	**-**	**-**
^{13}C field / kHz	-	14.4	-	-
^{15}N field / kHz	13.0	-	-	-
Shape	Tangent ^{1}H	Tangent ^{1}H	-	-
CP contact time / ms	0.9	0.5	-	-
INEPT delay (1/4J) / ms	-	-	2.2	1.8
INEPT refocusing delay / ms	-	-	2.2	1.4
**Transfer 2**	**NH (dipolar)**	**CH (dipolar)**	**NH (INEPT)**	**CH (INEPT)**
^{1}H field / kHz	80.0	80.0	**-**	**-**
^{13}C field / kHz	-	14.4	-	-
^{15}N field / kHz	13.0	-	-	-
Shape	Tangent ^{1}H	Tangent ^{1}H	-	-
CP contact time / ms	1.3	0.6	-	-
INEPT delay (1/4J) / ms	-	-	2.2	1.8
INEPT refocusing delay / ms	-	-	2.2	1.4
Carrier ^{1}H / ppm	4.8	4.8	4.8	4.8
Carrier ^{13}C / ppm	-	55	-	55
Carrier ^{15}N / ppm	117.5	-	117.5	117.5
**Figure n°**	-	S10B	**-**	S10D

3D spectra: 3D hNCAHA

Experiment	ORF7b^{AFL}	ORF7b^{ATV}	ORF7b^{SCHIWA}	ORF7b^{PYQKANDEM}
^{1}H Field / MHz	850	850	850	850
MAS frequency / kHz	100	100	100	100
Sample Temperature / °C	27	23 (estimated)	27	21
Rotor diameter / mm	0.7	0.7	0.7	0.7
Number of scans	16	64	16	96
Experimental time	1 d 14.9 h	7 d 2.9 h	1 d 14.9 h	4 d 12 h
t1 increment	80	80	80	54
Sweep width (t1) / ppm	30.8	25.0	49.7	20
Acquisition time (t1) / ms	15.1	18.5	9.3	15.7
t2 increment	80	80	80	54
Sweep width (t2) / ppm	80	80	80	55
Acquisition time (t2) / ms	2.3	2.3	2.3	2.3
t3 increment	2048	3072	2048	3072
Sweep width (t3) / ppm	46.7	46.7	46.7	46.7
Acquisition time (t3) / ms	25.8	38.7	25.8	38.7
^{1}H decoupling (swftppm) / kHz	10	10	10	10
^{13}C decoupling (WALTZ64) / kHz	5	5	5	5
^{15}N decoupling (WALTZ64) / kHz	5	5	5	5
MISSISSIPPI (120 ms) / kHz	20	20	20	20
Interscan delay / s	1.0	1.0	1.0	1.0
**Transfer 1**	**HN (dipolar)**	**HN (dipolar)**	**HN (dipolar)**	**HN (dipolar)**
^{1}H field / kHz	80.0	80.0	80.0	80.0
^{15}N field / kHz	15.3	13.7	13.3	11.6
Shape	Tangent ^{1}H	Tangent ^{1}H	Tangent ^{1}H	Tangent ^{1}H
CP contact time / ms	0.8	0.9	1.0	1.0
**Transfer 2**	**NC (dipolar)**	**NC (dipolar)**	**NC (dipolar)**	**NC (dipolar)**
^{15}N field / kHz	35.0	35.0	35.0	35.0
^{13}C field / kHz	64.3	62.2	61.6	61.9
Shape	Tangent ^{13}C	Tangent ^{13}C	Tangent ^{13}C	Tangent ^{13}C
CP contact time / ms	20	24	22	14
**Transfer 3**	**HC (dipolar)**	**HC (dipolar)**	**HC (dipolar)**	**HC (dipolar)**
^{1}H field / kHz	71.1	80.0	75.4	80.0
^{13}C field / kHz	19.2	12.7	17.9	13.9
Shape	Tangent ^{1}H	Tangent ^{1}H	Tangent ^{1}H	Tangent ^{1}H
CP contact time / ms	0.7	0.7	0.5	0.5
Carrier ^{1}H / ppm	4.8	4.8	4.8	4.7
Carrier ^{13}C / ppm	40	50	40	37.5

Carrier ^{15}N / ppm	110	117.5	110	120
**Figure n°**	S3A	S3B	S3C	S3D

3D spectra: 3D hCCH-TOBSY

Experiment	ORF7b^{AFL}	ORF7b^{ATV}	ORF7b^{SCHIWA}	ORF7b^{PYQKANDEM}
^{1}H Field / MHz	850	850	850	850
MAS frequency / kHz	100	100	100	100
Sample Temperature / °C	27	23 (estimated)	27	21
Rotor diameter / mm	0.7	0.7	0.7	0.7
Number of scans	16	64	16	96
Experimental time	38.9 h	7d 2.9 h	1d 14.9 h	4 d 12 h
t1 increment	80	80	80	54
Sweep width (t1) / ppm	80	80	80	20
Acquisition time (t1) / ms	2.3	2.3	2.3	15.7
t2 increment	80	80	80	54
Sweep width (t2) / ppm	80	80	80	55
Acquisition time (t2) / ms	2.3	2.3	2.3	2.3
t3 increment	2048	3072	2048	3072
Sweep width (t3) / ppm	46.7	46.7	46.7	46.7
Acquisition time (t3) / ms	25.8	38.7	25.8	38.7
^{1}H decoupling (swftppm) / kHz	10	10	10	10
^{13}C decoupling (WALTZ64) / kHz	5	5	5	5
MISSISSIPPI (120 ms) / kHz	20	20	20	20
Interscan delay / s	2	2	2	2
**Transfer 1**	**HC (dipolar)**	**HC (dipolar)**	**HC (dipolar)**	**HC (dipolar)**
^{1}H field / kHz	80.0	80.0	80.0	80.0
^{13}C field / kHz	19.2	12.7	17.9	13.9
Shape	Tangent ^{1}H	Tangent ^{1}H	Tangent ^{1}H	Tangent ^{1}H
CP contact time / ms	0.8	0.9	1.0	1.0
**TOBSY mixing**	**CC (TOBSY)**	**CC (TOBSY)**	**CC (TOBSY)**	**CC (TOBSY)**
^{13}C field / kHz	46.2	46.2	46.2	46.2
Mixing scheme	$C9_{39}^{1}$	$C9_{39}^{1}$	$C9_{39}^{1}$	$C9_{39}^{1}$
Shape	Post_element.720	Post_element.720	Post_element.720	Post_element.720
Mixing time / ms	15.2	15.2	15.2	15.2
**Transfer 3**	**CH (dipolar)**	**CH (dipolar)**	**CH (dipolar)**	**CH (dipolar)**
^{1}H field / kHz	71.1	80.0	75.4	80.0
^{13}C field / kHz	19.2	12.7	17.9	13.9
Shape	Tangent ^{1}H	Tangent ^{1}H	Tangent ^{1}H	Tangent ^{1}H
CP contact time / ms	0.7	0.7	0.5	0.5
Carrier ^{1}H / ppm	4.8	4.8	4.8	4.7
Carrier ^{13}C / ppm	40	50	40	37.5
**Figure n°**	4E	S8C	S9C	S10C

Further 2D hCH spectra

Experiment	ORF7b^{AFL} in ERGIC	ORF7b^{AFL} in d82-POPC	E-cadTM^{AGPL}
^{1}H Field / MHz	1200	1200	850
MAS frequency / kHz	100	100	100
Sample Temperature / °C	22	20	27
Rotor diameter / mm	0.7	0.7	0.7
Number of scans	64	64	64
Experimental time	4.3 h	4.7 h	4.1 h
t1 increment	180	196	170
Sweep width (t1) / ppm	130	130	100
Acquisition time (t1) / ms	2.3	2.5	4.0
t2 increment	3072	3072	3072
Sweep width (t2) / ppm	46.3	46.3	46.7
Acquisition time (t2) / ms	27.6	27.6	38.7
^{1}H decoupling (swftppm) / kHz	10	10	10
^{13}C decoupling (WALTZ64) / kHz	5	5	5
^{15}N decoupling (WALTZ64) / kHz	5	5	-
MISSISSIPPI (120ms) / kHz	20	20	20
Interscan delay / s	1.2	1.2	1.2
**Transfer 1**	**HC (dipolar)**	**HC (dipolar)**	**HC (dipolar)**
^{1}H field / kHz	80.0	77.4	80.0
^{13}C field / kHz	17.6	20.0	15.8
Shape	Tangent ^{1}H	Tangent ^{1}H	Tangent ^{1}H
CP contact time / ms	0.7	0.6	0.6
**Transfer 2**	**CH (dipolar)**	**CH (dipolar)**	**CH (dipolar)**
^{1}H field / kHz	80.0	77.4	80.0
^{13}C field / kHz	17.6	20.0	15.8
Shape	Tangent ^{1}H	Tangent ^{1}H	Tangent ^{1}H
CP contact time / ms	0.7	0.7	0.5
Carrier ^{1}H / ppm	4.8	4.8	4.8
Carrier ^{13}C / ppm	72.5	77.2	52.5
Carrier ^{15}N / ppm	120	120	-
**Figure n°**		S11A (red)	S13

Further 2D hCH spectra

Experiment	$ORF7b^{FC}$	ERGIC (INEPT)	$ORF7b^{L:F}$	$ORF7b^{F}$:E-$cadTM^{L}$
^{1}H Field / MHz	1200	1200	1200	1200
MAS frequency / kHz	100	100	100	100
Sample Temperature / °C	20	23	23 (estimated)	21
Rotor diameter / mm	0.7	0.7	0.7	0.7
Number of scans	64	64	64	64
Experimental time	2.2 h	4.1 h	4.3 h	4.3 h
t1 increment	90	170	180	180
Sweep width (t1) / ppm	130	100	130	130
Acquisition time (t1) / ms	1.1	2.8	2.3	2.3
t2 increment	3072	3072	3072	3072
Sweep width (t2) / ppm	46.3	46.3	46.3	46.3
Acquisition time (t2) / ms	27.6	27.6	27.6	27.6
^{1}H decoupling (swftppm) / kHz	10	10	10	10
^{13}C decoupling (WALTZ64) / kHz	5	5	5	5
^{15}N decoupling (WALTZ64) / kHz	5	-	5	5
MISSISSIPPI (120ms) / kHz	20	20	20	20
Interscan delay / s	1.2	1.2	1.2	1.2
**Transfer 1**	**HC (dipolar)**	**HC (INEPT)**	**HC (dipolar)**	**HC (dipolar)**
^{1}H field / kHz	80.0	-	80.0	80.0
^{13}C field / kHz	17.5	-	13.1	16.6
Shape	Tangent ^{1}H	-	Tangent ^{1}H	Tangent ^{1}H
CP contact time / ms	0.6	-	0.6	0.6
INEPT delay (1/4J) / ms	-	1.8	-	-
INEPT refocusing delay / ms	-	1.4	-	-
**Transfer 2**	**CH (dipolar)**	**CH (INEPT)**	**CH (dipolar)**	**CH (dipolar)**
^{1}H field / kHz	80.0	-	80.0	80.0
^{13}C field / kHz	17.5	-	13.1	16.6
Shape	Tangent ^{1}H	-	Tangent ^{1}H	Tangent ^{1}H
CP contact time / ms	0.7	-	0.7	0.7
INEPT delay (1/4J) / ms	-	1.8	-	-
INEPT refocusing delay / ms	-	1.4	-	-
Carrier ^{1}H / ppm	4.9	4.9	5.3	4.8
Carrier ^{13}C / ppm	72.7	57.9	83	77.5
Carrier ^{15}N / ppm	120	-	120	120
**Figure n°**	S13B (red)	S13C	5B (teal)	6D (teal)

hChH-MIRROR spectra

Experiment	ORF7b^{AFL}	ORF7b^{AFL} in d82 POPC	ERGIC
^{1}H Field / MHz	1200	1200	850
MAS frequency / kHz	100	100	100
Sample Temperature / °C	22	20	27
Rotor diameter / mm	0.7	0.7	0.7
Number of scans	320	320	320
Experimental time	11.2 h	11.2 h	11.2 h
t1 increment	90	90	90
Sweep width (t1) / ppm	60	60	60
Acquisition time (t1) / ms	2.5	2.5	3.5
t2 increment	3072	3072	3072
Sweep width (t2) / ppm	46.3	46.3	46.3
Acquisition time (t2) / ms	27.6	27.6	38.7
^{1}H decoupling (swftppm) / kHz	10	10	10
^{13}C decoupling (WALTZ64) / kHz	5	5	5
MISSISSIPPI (120 ms) / kHz	20	20	20
Interscan delay / s	1.2	1.2	1.2
**Transfer 1**	**HC (dipolar)**	**HC (dipolar)**	**HC (dipolar)**
^{1}H field / kHz	80.0	77.4	80.0**
^{13}C field / kHz	17.6	20.0	13.8**
Shape	Tangent ^{1}H	Tangent ^{1}H	Tangent ^{1}H
CP contact time / ms	0.7	0.6	0.5**
**Transfer 3**	**CH (dipolar)**	**CH (dipolar)**	**CH (dipolar)**
^{1}H field / kHz	80.0	77.4	80.0**
^{13}C field / kHz	17.6	20.0	13.8**
Shape	Tangent ^{1}H	Tangent ^{1}H	Tangent ^{1}H
CP contact time / ms	0.7	0.7	0.6**
**MIRROR mixing**	**HH (MIRROR)**	**HH (MIRROR)**	**HH (MIRROR)**
^{13}C field / kHz	6.6*	6.6*	4.7**
Mixing time / ms	50	50	50
Carrier ^{13}C for mixing / ppm	27.0	20.9	26.9
Carrier ^{1}H / ppm	4.8	4.8	4.9
Carrier ^{13}C / ppm	42.5	42.2	42.6
**Figure n°**	4E	S13A (blue)	S13C

* The RF-field of the ^{13}C pulse of the reverse MIRROR mixing scheme corresponds to the resonance frequency difference of $^{1}H^{\gamma}$ of Leu and aromatic ^{1}H-s of the Phe residues
** These parameters were copied from a protein sample because they could not be optimized for the lipids.

hChH-MIRROR spectra

**Experiment**	**ORF7b^{FC}**	**ORF7b$^{L:F}$**	**ORF7b^{F}:E-cadTM^{L}**
^{1}H Field / MHz	1200	1200	1200
MAS frequency / kHz	100	100	100
Sample Temperature / °C	20	23 (estimated)	19
Rotor diameter / mm	0.7	0.7	0.7
Number of scans	320	1280	1280
Experimental time	11.2 h	1 d 20.8 h	1 d 20.8 h
t1 increment	90	90	90
Sweep width (t1) / ppm	60	60	60
Acquisition time (t1) / ms	2.5	2.5	2.5
t2 increment	3072	3072	3072
Sweep width (t2) / ppm	46.3	46.3	46.3
Acquisition time (t2) / ms	27.6	27.6	27.6
^{1}H decoupling (swftppm) / kHz	10	10	10
^{13}C decoupling (WALTZ64) / kHz	5	5	5
MISSISSIPPI (120ms) / kHz	20	20	20
Interscan delay / s	1.2	1.2	1.2
**Transfer 1**	**HC (dipolar)**	**HC (dipolar)**	**HC (dipolar)**
^{1}H field / kHz	80.0	80.0	80.0
^{13}C field / kHz	17.5	13.1	16.6
Shape	Tangent ^{1}H	Tangent ^{1}H	Tangent ^{1}H
CP contact time / ms	0.6	0.6	0.6
**Transfer 3**	**CH (dipolar)**	**CH (dipolar)**	**CH (dipolar)**
^{1}H field / kHz	80.0	80.0	80.0
^{13}C field / kHz	17.5	13.1	16.6
Shape	Tangent ^{1}H	Tangent ^{1}H	Tangent ^{1}H
CP contact time / ms	0.7	0.7	0.7
**MIRROR mixing**	**HH (MIRROR)**	**HH (MIRROR)**	**HH (MIRROR)**
^{13}C field / kHz	6.3*	6.6*	6.3*
Mixing time / ms	50	50	50
Carrier ^{13}C for mixing / ppm	27.5	24.0	26.5
Carrier ^{1}H / ppm	4.9	5.0	4.8
Carrier ^{13}C / ppm	42.3	42.1	42.2
**Figure n°**	S13B (blue)	5B (magenta)	6D (magenta)

* The RF-field of the ^{13}C pulse of the reverse MIRROR mixing scheme corresponds to the resonance frequency difference of $^{1}H^{\gamma}$ of Leu and aromatic ^{1}H-s of the Phe residues

HH-spectrum

Experiment	E-cadTM^{L}: ORF7b^{F}
^{1}H Field / MHz	1200
MAS frequency / kHz	100
Sample Temperature / °C	27
Rotor diameter / mm	0.7
Number of scans	16
Experimental time	1.8 h
t1 increment	200
Sweep width (t1) / ppm	12.0
Acquisition time (t1) / ms	6.9
t2 increment	3072
Sweep width (t2) / ppm	46.3
Acquisition time (t2) / ms	27.6
presaturation / kHz	0.15
presaturation length / ms	500
Interscan delay / s	1.2
**Spindiffusion mixing**	**HH (SD)**
Mixing time / ms	150
Carrier ^{1}H / ppm	5.0
**Figure n°**	S13D

Table S8: List of selectively labeled NMR samples

Residues not labelled with ^{13}C and ^{15}N are per-deuterated except at exchangeable sites. ERGIC was used in natural isotopic abundance.

Name	^{13}C ^{15}N residues	Lipids
$ORF7b^{ATV}$	Ala, Thr, Val	ERGIC
$ORF7b^{AFL}$	Ala, Leu, Phe	ERGIC
$ORF7b^{AFL}$	Ala, Leu, Phe	d82-POPC
$ORF7b^{SCHIWA}$	Ala, Cys, His, Ile, Ser, Trp	ERGIC
$ORF7b^{PYQKANDEM}$	Ala, Asn, Asp, Gln, Glu, Lys, Met, Pro, Tyr	ERGIC
$ORF7b^{F}$	Phe	ERGIC
$ORF7b^{L}$	Leu	ERGIC
$ORF7b^{FC}$	Phe, Cys	ERGIC
$E\text{-}cadTM^{L}$	Leu	ERGIC
ERGIC lipids (no protein)	-	ERGIC
